# Use of Composite Protein Database including Search Result Sequences for Mass Spectrometric Analysis of Cell Secretome

**DOI:** 10.1371/journal.pone.0121692

**Published:** 2015-03-30

**Authors:** Jihye Shin, Gamin Kim, Mohammad Humayun Kabir, Seong Jun Park, Seoung Taek Lee, Cheolju Lee

**Affiliations:** 1 Center for Theragnosis, BRI, Korea Institute of Science and Technology, Seoul 136–791, Korea; 2 Department of Biochemistry, College of Life Science and Biotechnology, Yonsei University, Seoul 120–749, Korea; 3 Department of Pathology, Yonsei University College of Medicine, Seoul 120–752, Korea; 4 Department of Biomolecular Science, University of Science and Technology, Daejeon 305–333, Korea; CHA University, KOREA, REPUBLIC OF

## Abstract

Mass spectrometric (MS) data of human cell secretomes are usually run through the conventional human database for identification. However, the search may result in false identifications due to contamination of the secretome with fetal bovine serum (FBS) proteins. To overcome this challenge, here we provide a composite protein database including human as well as 199 FBS protein sequences for MS data search of human cell secretomes. Searching against the human-FBS database returned more reliable results with fewer false-positive and false-negative identifications compared to using either a human only database or a human-bovine database. Furthermore, the improved results validated our strategy without complex experiments like SILAC. We expect our strategy to improve the accuracy of human secreted protein identification and to also add value for general use.

## Introduction

Proteins secreted from cells and tissues are likely to enter body fluids and may be essential in several processes such as differentiation, invasion, metastasis and angiogenesis of cancers by regulating cell-to-cell and cell-to-extracellular matrix interactions [1]. Cell line secretomes can be analyzed to identify proteins with the potential to enter the circulatory system as an alternative to direct analysis of serum due to the wide concentration range and complexity of serum proteins [2]. Hundreds of articles concerning in-depth profiling of secretomes have been published owing to advancements in mass spectrometry (MS) and proteomic techniques in recent years [3]. However, the main drawbacks of analyzing secretomes involve contamination by intracellular proteins and fetal bovine serum (FBS) proteins used in cell culture media [4]. In a typical shotgun LC-MS/MS experiment, FBS contaminants are sometimes difficult to discriminate from the proteins truly secreted by cells because of shared peptide sequences between species. Nevertheless, it is important to distinguish FBS proteins from human secreted proteins for selecting marker candidates since FBS proteins themselves are present predominantly in secreted proteins. Only a few studies have attempted to isolate secretome from bovine serum proteins [5–8], and many research publications have described it incorrectly.

Herein, we have characterized contaminants derived from FBS in shotgun LC-MS/MS by investigating the following: (1) How much of the FBS proteins remain in the conditioned media (CM)? (2) Can identification of human proteins be improved by only manipulating sequence databases? (3) What are the factors that lead to false-positive peptide spectrum matches (PSM)? (4) Is our strategy more effective than other applications? A series of experiments were performed to answer these questions. We first identified the amount of residual FBS proteins, yielding a list of 199 proteins through FBS profiling. Afterward, we compared our secretome raw MS data against a composite database named HFDB which comprised of human UniProt sequences and the aforementioned FBS search result sequences. The results using HFDB were more reliable with more true positive matches compared to using human only or human-bovine databases, and was further confirmed via SILAC experiment. Our strategy produced improved results without the need for multifaceted experiments to isolate FBS proteins from secretome. From our results, we expect that it will be helpful to consider the contaminants stemming from FBS in secretome analysis. Therefore, comparing LC-MS/MS data against the appropriate database will maximize correct identification.

## Experimental Details

### FBS and culture plates

Sterile-filtered FBS originating from the United States (endotoxin level ≤ 5 EU/mL, hemoglobin level ≤ 10 mg/dL) was purchased from Gibco Inc., (cat. # 16000, Rockville, MD). Cell culture plates were polystyrene treated Nunclon Delta types obtained from Nunc (cat. # 168381, Thermo Fisher Scientific Inc., Roskilde, Denmark). FBS was diluted 10 times with lysis buffer consisting of 8 M urea, 75 mM NaCl, 50 mM Tris (pH 8.2) and was stored at -80°C until use.

### Preparation of Secretome

Calu-1, one of the lung cancer cell lines, was obtained from the American Type Culture Collection. Cells were cultured in RPMI1640 (Gibco, Rockville, MD) supplemented with 10% FBS (Gibco, Rockville, MD), 1% penicillin and streptomycin (Gibco, Rockville, MD) at 37°C in a humidified 95% air, 5% CO_2_ incubator. Cells were grown to approximately 70% confluence (approximately 1.6 × 10^7^ cells) in 150 mm culture dishes (Nunc, Naperville, IL). The cell monolayer was carefully rinsed three times with serum-free medium (SFM) at room temperature. The cells were then incubated in the SFM with 1% penicillin and streptomycin at 37°C for 12 h. The 12-hour incubation time was chosen to minimize release of cytosolic proteins into surrounding medium due to cell death. After incubation, the conditioned media was carefully collected and 2 mM PMSF and 1 mM EDTA were added as protease inhibitors. Floating cells and cellular debris were removed by centrifugation (400 ×*g*, 10 min, 4°C), followed by sterile filtration (pore size: 0.22 μm, Millipore, MA). The media were concentrated by ultrafiltration using ‘Amicon Ultra-15’ centrifugal filter devices (Millipore, MA). Secreted proteins in the media were transferred to lysis buffer consisting of 8 M urea, 75 mM NaCl, 50 mM Tris (pH 8.2).

### Preparation of contaminants without secretome

Cell-free polystyrene-plates (150 mm) containing RPMI1640 and DMEM media supplemented with 10% FBS were incubated for 2 days. The plates were washed with SFM three times and incubated for 12 h. The media were collected and concentrated 250 times (50 mL to 200 μl) by ultrafiltration as described above. To each of the empty plates, 400 μL of lysis buffer was added and the surfaces of the plates were physically scraped. The lysis buffers from the cell-free conditioned media were collected and analyzed separately. Protein concentration was determined by a standard Bradford protein assay (Bio-Rad, Richmond, CA), and all protein samples were stored at -80°C until use.

### SILAC

HCT-116 cells were cultured in SILAC RPMI medium (RPMI-1640 media deficient in L-lysine and L-arginine; Thermo Scientific, San Jose, CA) supplemented with 10% dialyzed fetal bovine serum (FBS) (dialyzed by ultrafiltration, Sigma-Aldrich), 1× penicillin/streptomycin (Gibco, Rockville, MD). For SILAC labeling, arginine (Arg) and lysine (Lys) were added in heavy amino acids (Arg10; Lys8) forms to a final concentration of 253.7 μg/mL for Arg and 55.2 μg/mL for Lys. [L-^13^C_6_,^15^N_4_]-Arg (Arg10) and [L-^13^C_6_,^15^N_2_]-Lys (Lys8) were purchased from Cambridge isotope. HCT-116 cells were grown in SILAC medium for 8 passages. Secretome from SILAC-labeled cells were collected after 24 h using serum-free SILAC media, at which α-tubulin, a cytosolic protein was scarcely detected in the media.

### In-Solution Digestion

Proteolytic digestion of proteins and mass spectrometric analysis were similar to the method described previously [9]. The protein samples were reduced with 10 mM DTT (Sigma, St Louis, MO) at 30°C for 1 h and alkylated with 14 mM iodoacetamide (Sigma, St Louis, MO) at 25°C for 1 h in the dark. The samples were diluted five times with 50 mM Tris (pH 8.2) to decrease the urea concentration to 1.6 M and CaCl_2_ (Sigma, St Louis, MO) was added up to a final concentration of 1 mM. The protein mixtures were digested by sequencing grade modified trypsin (Promega, Madison, WI) at 37°C for 16 h. The ratio of enzyme to protein was 1:50. The tryptic digests were directly used after desalting or further separated according to isoelectric point by using an OFFGEL fractionator (Agilent technology, Santa Clara, CA). The separated peptides were collected into 14 fractions. Peptide samples were desalted with a C_18_ spin column (Thermo Scientific, San Jose, CA) prior to LC-MS/MS, dried *in vacuo*, and kept at -80°C for subsequent analysis.

### LC-MS/MS

The peptide samples were reconstituted in 0.4% acetic acid. An aliquot (~1 μg) was then injected into a reversed-phase Magic C18aq column (15 cm × 75 μm, 200Ǻ) on an Agilent 1200 HPLC system (Agilent technology, Santa Clara, CA). The column was pre-equilibrated with 95% buffer A (0.1% formic acid in water) and 5% buffer B (0.1% formic acid in acetonitrile). The peptides were eluted at a flow rate of 0.4 μL/min across the analytical column with 5–40% gradient buffer B for 90 min for OFFGEL fractions, or 5–40% gradient buffer B for 120 min for unfractionated samples. The HPLC system was coupled to an LTQ-XL mass spectrometer (Thermo Scientific, San Jose, CA). ESI spray voltage was set to 1.9 kV, capillary voltage to 30 V and the temperature of the heated capillary to 250°C. MS survey was scanned from 300 to 2,000 m/z, and followed by three data-dependent MS/MS scans with the following parameters: isolation width, 1.5 m/z; normalized collision energy, 25%; dynamic exclusion duration, 180 s. All data were acquired using Xcalibur software v2.0.7. All analyses were conducted in duplicate runs to exhibit nearly identical retention time, and to confirm a high level of reproducibility for the LTQ instrument.

### Analysis of mass spectrometric data

The acquired MS/MS spectra were compared against various databases using SEQUEST in Proteome Discoverer 1.4 (Thermo Fisher Scientific, version 1.4.0.288). Proteome Discoverer software performs automated statistical analysis of the search result. The databases used were human database (HuDB, 88,304 entries), bovine database (BoDB, 24,235 entries), human-bovine database (HBDB, 112,539 entries) and human-FBS database (HFDB, 88,503 entries). HuDB and BoDB contained the Uniport protein sequences released as of 2013.07. HBDB was constructed by merging BoDB to HuDB. HFDB was constructed by appending 199 bovine protein sequences to HuDB. The 199 bovine sequences were bovine UniProt sequences identified earlier by LC-MS/MS analysis of FBS. Finally, an additional 20 sequences representing proteases (trypsin, chymotrypsin, Arg-C, Lys-C, Asp-N, and their fragments) and mass standards (yeast enolases which are regularly used for instrument tuning in our laboratory) were appended to each database. The following options were applied during searches: enzyme specificity, trypsin; number of missed cleavages, two; precursor mass tolerance, ±2 Da; fragment mass tolerance, ± 0.5 Da; fixed modification, carbamidomethylation at cysteine (+57.02 Da); variable modification, oxidation at methionine (+15.99 Da). For the analysis of SILAC samples, variable modifications of [L-^13^C_6_,^15^N_4_]-Arg (+10.01 Da) and [L-^13^C_6_,^15^N_2_]-Lys (+8.01 Da) were permitted. Arg-Pro conversion in eukaryotes was taken into consideration by an additional variable modification to proline (+6.01 Da). Percolator, which uses semi-supervised learning and a decoy database search strategy to distinguish between correct and incorrect PSMs identified by a database search algorithm,[10] was used to calculate q value as a statistical confidence measure for each PSM. We filtered the output with q < 0.01. The final data were exported from the Proteome Discoverer to a spreadsheet format using MS-Excel. During export, a 1% FDR cutoff was applied. The peptide and protein data were further filtered using the following criteria: high peptide confidence, top peptide rank filter, XCorr threshold set differently according to charge state (+1: 1.5, +2: 2, +3: 2.25, +4: 2.5, +5: 2.75, +6: 3, +7: 3.2 and >+7: 3.4), minimal number of unique peptides per protein: one; protein score threshold: 10, and protein search engine rank of 1. All MS files are available from the PeptideAtlas database (dataset identifier: PASS00579; http://www.peptideatlas.org/PASS/PASS00579).

## Results and Discussion

During cell culture, the protein concentration of 10% FBS in growth medium is 4–5 mg/mL. Despite several washings, bovine proteins are difficult to remove completely due to adherence to the polystyrene-plate surfaces and to the cultured human cells. To quantify residual FBS in CM, identical 150 mm polystyrene-plates (~180 cm^2^) were incubated for 2 days with RPMI1640 and DMEM supplemented with 10% FBS. After three washings and subsequent incubation with serum-free media (SFM) for 12 h, the concentration of FBS in SFM was measured as 100~160 ng/mL. The amount of proteins adhered to the polystyrene-plates was found to be 300~690 ng per plate, assuming that scraping the plates completely detached the adhered proteins. Although >99.9% FBS was washed out by rinsing, the result implies that FBS contaminants are difficult to ignore owing to the abundance above the MS detection limits. The amount of cell-secreted proteins was measured as 2.0~5.0 μg/mL. Simple arithmetic indicates that the FBS contaminant is expected to be 5~10% in secretome.

**Fig 1:**
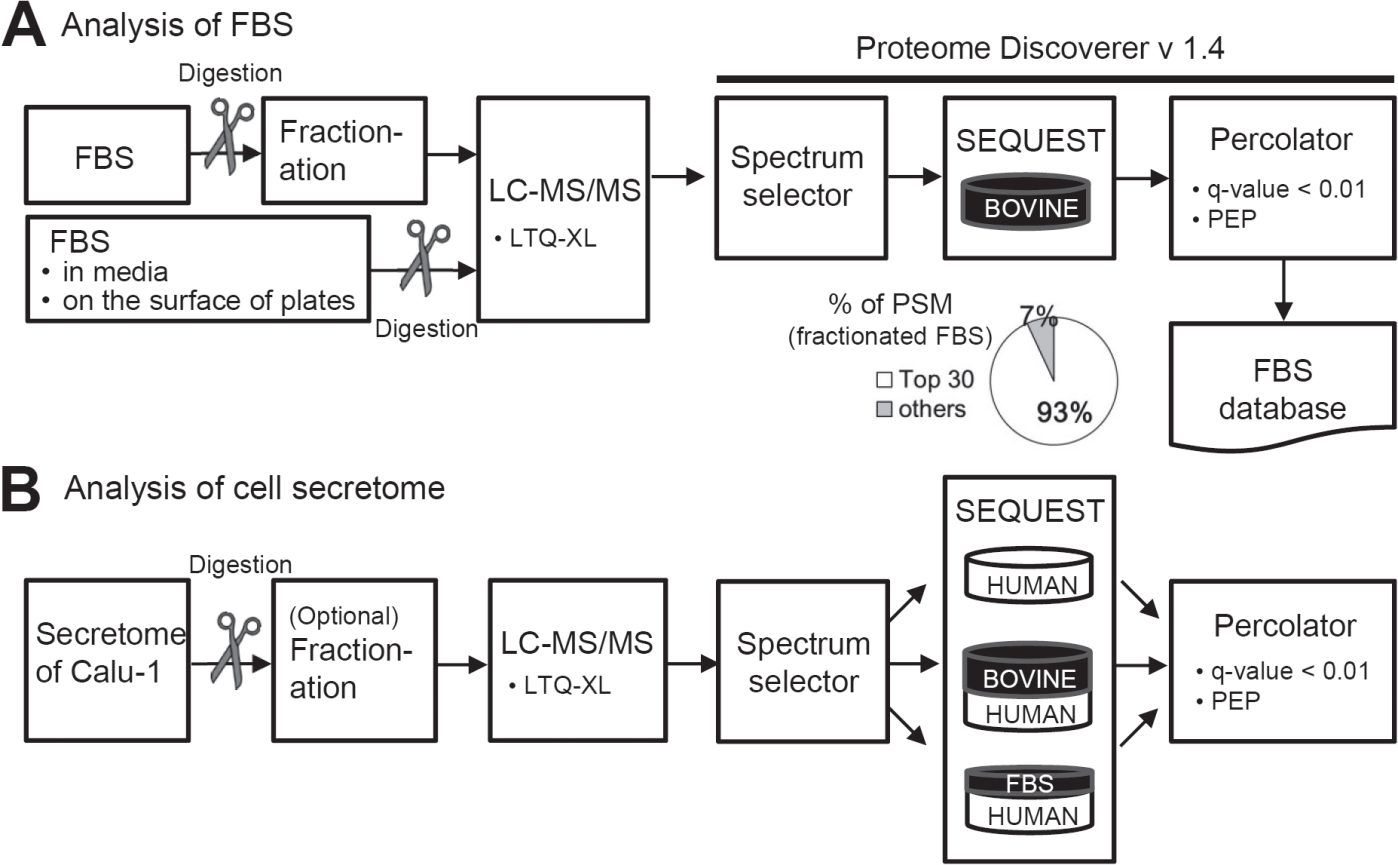
Schematic workflow for the proteome analysis of (A) fetal bovine serum (FBS) and (B) cell secretome. (A) FBS by itself as well as the residual FBS in the conditioned media or on the surface of the culture plate were analyzed. MS/MS data were compared against a bovine sequence database and the resultant protein list was used for the construction of an FBS database. (B) MS/MS data from cell secretome analysis were compared against various databases including human, human plus bovine and human plus FBS databases.

To characterize FBS proteins, the FBS was digested with trypsin and the resultant tryptic peptides were analyzed by LC-MS/MS after OFFGEL fractionation. Residual FBS in SFM and adhered to the plate surfaces were also analyzed without fractionation. As shown in Fig. 1A, the raw MS/MS data acquired from FBS analysis were compared against the UniProt Bovine database (24,235 entries). 17534 spectra were matched to 845 peptides, and 193 protein groups were identified. The top 30 proteins with the highest PSM numbers accounted for ~93% of all PSMs. In addition, 30 and 7 proteins were identified from the analysis of residual FBS in SFM and the plate surfaces, respectively. A total of 199 FBS proteins were identified from all approaches (S1 Table). Human cell secretome is likely to be contaminated by these bovine proteins over the course of a typical secretome preparation. Therefore, we have constructed a new database with the bovine UniProt sequences of these 199 proteins and designated it as the FBS database. The top 30 as well as the residual FBS proteins are designated as major contaminants hereafter.

With the results of FBS profiling, we looked into how to maximize true positive identifications from secretome analysis. SFM from Calu-1 cells was collected and analyzed through an identical process (Fig. 1B). The MS/MS raw data were compared against each of the three databases: HuDB, HBDB and HFDB, and a total of 3383, 3564 and 3577 PSMs were generated (S2 Table). The number of PSMs identified using the databases compounded with bovine protein sequences (HFDB and HBDB) was greater than with HuDB, presumably because of the presence of considerable FBS contaminants in the secretome. Therefore, using conventional HuDB in secretome analysis may lead to a number of false-negatives and may also include false-positive identifications.

**Fig 2:**
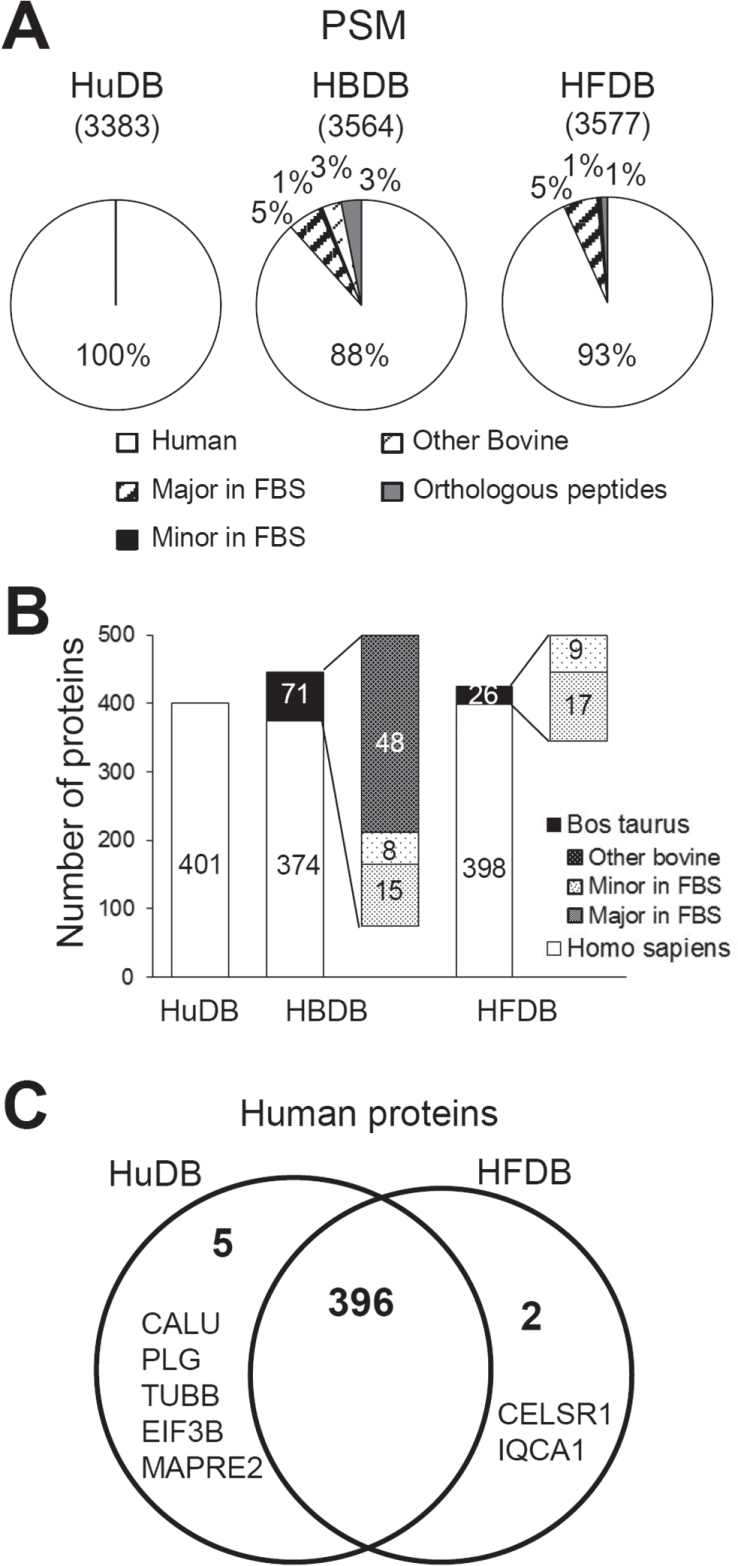
Search results of MS/MS data from Calu-1 secretome analysis vary depending on the database used. (A) PSMs are classified based on their species specificity. The classifications are PSMs belonging to: the top 30 FBS proteins or the proteins identified from residual FBS in the media or on the surface of the plate (Major in FBS), other FBS proteins (Minor in FBS), bovine proteins not identified by FBS analysis (Other bovine), and none of the species exclusively due to identical sequences (Orthologous peptides). (B) The number of identified proteins. Bovine proteins are subgrouped as in (A). (C) Venn diagram showing human proteins among the identified proteins.

Subsequently, we focused on the species-specificity of the peptides and proteins identified from the secretome and the way PSMs for each MS/MS spectrum changed depending on the search database used. The major bovine contaminants comprised about 5% of all PSMs while the other contaminants constituted only 1% according to the HFDB search result (Fig. 2A). This is highly consistent with not only the result of FBS analysis where the top 30 proteins occupied nearly 93% of all PSMs as shown in Fig. 1A, but also with the expectation that the FBS contaminants would account for around 5~10% of the secretome. When HBDB was used, the PSMs corresponding to orthologous peptides increased to 3%. Other 3% PSMs belonged to bovine proteins which had not been identified in FBS profiling. If a bovine protein in FBS cannot be identified even after deep-down analysis, then the protein is unlikely to be identified from a sample consisting mainly of human secreted proteins and a little residual FBS contaminant. Therefore, as previously revealed by other researchers [11,12], using a larger database in an attempt to decrease false-negatives seems to yield more false-positives. Similarly, Fig. 2B presents the number of protein groups identified from searching different databases. Though the number of human protein groups identified in HuDB and HFDB search results was similar, the proteins identified solely in each database (5 from HuDB and 2 from HFDB searches) were quite noteworthy (Fig. 2C and S1 Fig.). The PSMs of the 5 HuDB proteins were mostly orthologous peptide sequences, and thus crossed over to bovine protein groups in HFDB search results (S1B Fig.). However, the PSMs of 2 HFDB proteins were all unique peptides and the corresponding spectra did not match any PSMs in the HuDB search result (S1C Fig.). Conversely, 26 bovine proteins were identified in HFDB searches with 242 PSMs (Fig. 2B), many which were matched to human-bovine-shared peptides in the HuDB search. A few examples of false positive PSMs by HuDB are shown in S2 Fig. On the other hand, 71 bovine proteins were identified in the HBDB search, among which were 48 proteins comprising 3% PSMs never detected in FBS analysis. It seems that HBDB search results contained many false-positive identifications, since we determined that even minor bovine contaminants occupied less than 1% of the secretome. Thus, using a larger database remains challenging due to increased search space and an increased potential for false positives [13]. We conclude that the way to maximize correct identification of human secreted proteins is to compromise on the shrinkage of database size by inclusion of sequence entries for only the possible contaminants in the database.

**Fig 3:**
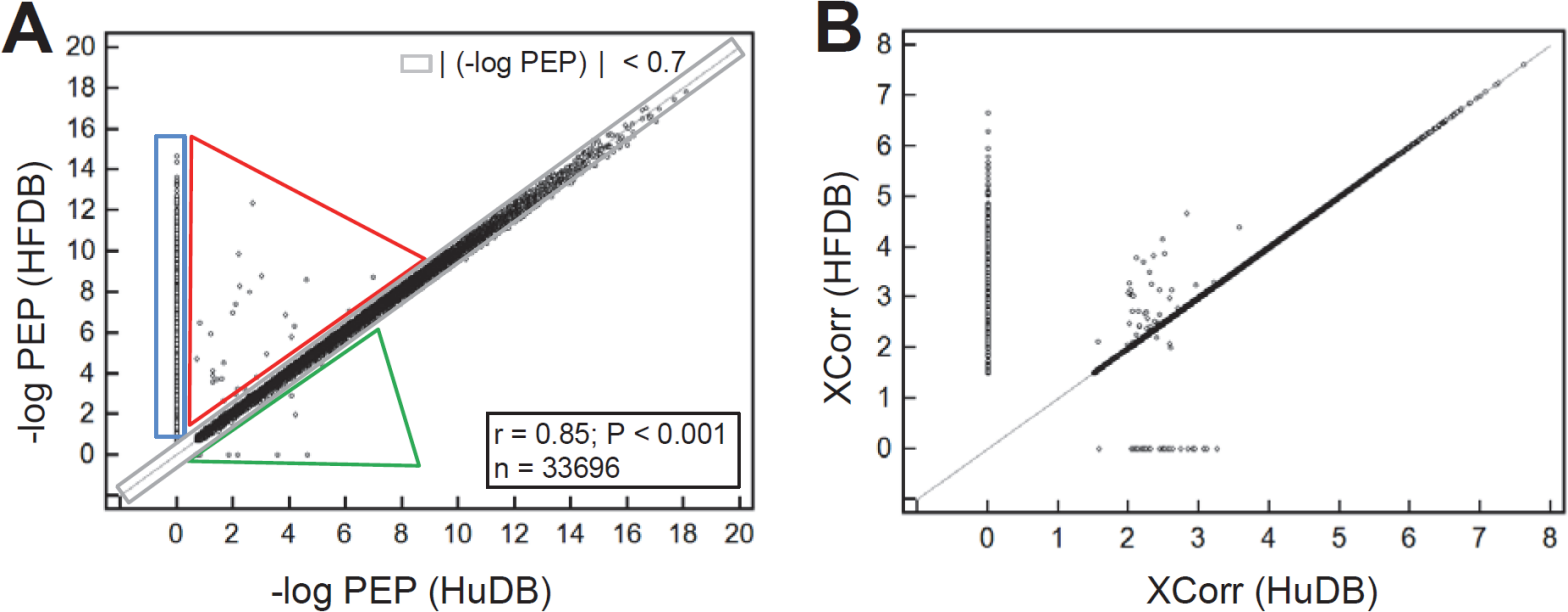
Distribution of posterior error probability (PEP) and Xcorr values for PSMs of Calu-1 secretome fractionated by OFFGEL fractionator. (A) -logPEP values are compared between each pair of peptide-spectrum matches (PSMs). The region where |Δ(-logPEP)| < 0.7 between HuDB and HFDB search results is marked grey. Spectra in blue area have PSMs in the HFDB, but not in HuDB search result. Spectra in red area have higher -logPEP value (more than 0.7) for the PSMs of HFDB than for HuDB search results. The reverse is true for the spectra in green area. (B) Distribution of XCorr values for HFDB vs. HuDB search results.

Results from the secretome fractionated by OFFGEL are similar to those of unfractionated samples and support our notion more clearly (S3 Table). The posterior error probability (PEP) value, which is known as the local false discovery rate and measures the probability of error for a single PSM (see reference [14]), is plotted between HFDB and HuDB search results. In Fig. 3A, ~93% of all PSMs (31418 out of 33696 PSMs) are located near the diagonal axis where |Δ(-logPEP)|< 0.7 (grey area in Fig. 3A). Spectra in the blue area (2197 PSMs) have PSMs in HFDB, but not in the HuDB search results. Surprisingly, 28 spectra in the red area were matched to human proteins in HuDB, but to bovine proteins in HFDB. It should be noted that these spectra have lower error probabilities for the PSM of HFDB than HuDB. This implies that matches of these 28 spectra to human sequences in the HuDB search might be false positive due to a lack of corresponding sequences in the database. Most of the PSMs in the green area had very high error probabilities and of those, only 4 PSMs PEP values were < 0.01. A similar pattern was observed in the plot of XCorr values from the HuDB and HFDB searches (Fig. 3B). Taken together, the analysis of the fractionated sample further supports that it is more reliable to use our HFDB to search secretome MS/MS data.

**Fig 4:**
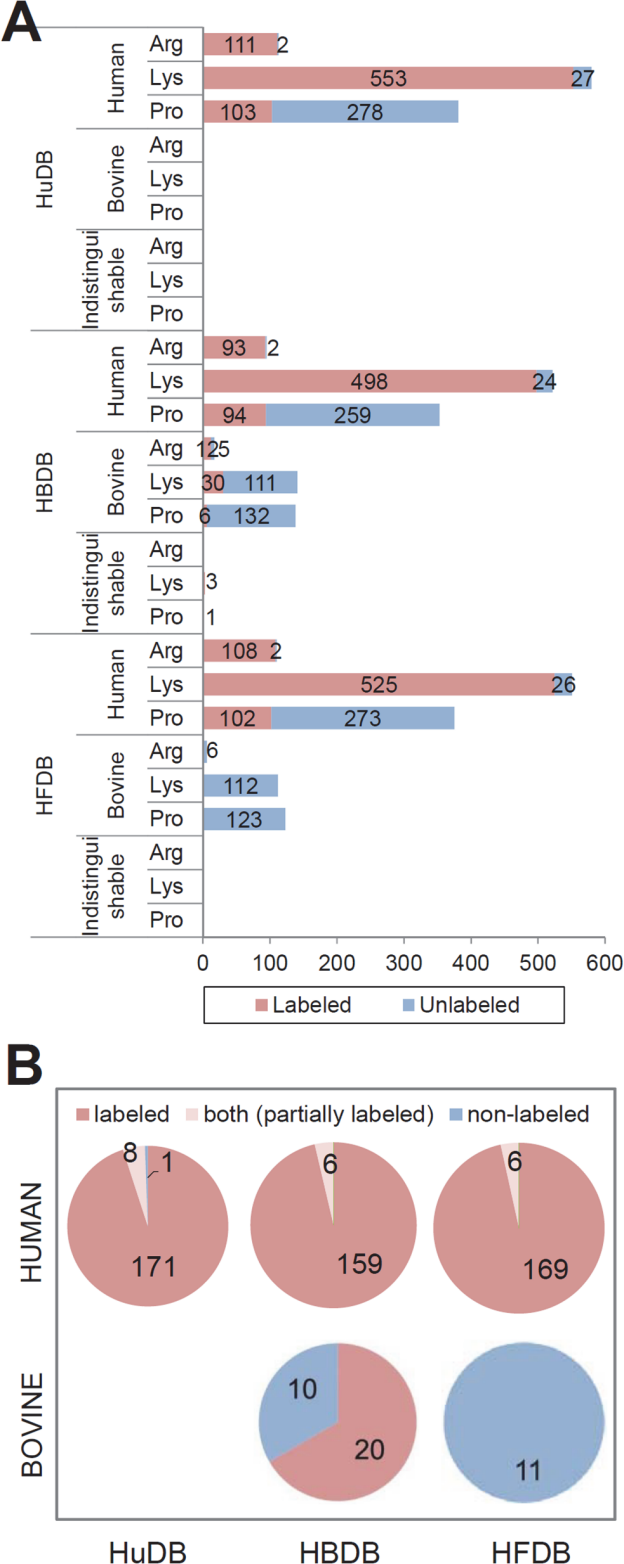
Search results of MS/MS data from SILAC-labeled secretome of HCT-116. (A) Mass spectrometric data were searched against HuDB, HBDB and HFDB databases, and then arginine, lysine, and proline residues in all PSMs were counted and classified into isotope-labeled (red) and unlabeled (blue) residues. (B) Pie-charts showing the number of proteins classified as human and bovine. Labeled: identification from all labeled peptides; partially labeled: identification from both labeled and unlabeled peptides; non-labeled: identification from unlabeled peptides only.

Distinguishing human proteins from bovine contaminants in secretomes is experimentally enabled in the SILAC-based approach. Supplementation of isotopically labeled amino acids in serum-containing medium results in the labeling of human cell-originated proteins while leaving FBS proteins unlabeled. Thus, even peptides with identical sequences originating from different species can be distinguished. Colzani et al. [6] and Villarreal et al. [8] reported results from the analyses of SILAC-based cell secretomes. The results are comparable to ours in that 5% to 20% of the PSMs were derived from FBS or false positives [6,8]. We also confirmed whether the proteins identified by HFDB as having bovine origins were true identifications by performing SILAC experiments. Mass spectrometric data of the SILAC-labeled secretome of HCT-116 were compared against HuDB, HBDB and HFDB, generating a total of 513, 582 and 591 PSMs, respectively. Arginine, lysine, and proline residues in all PSMs were then counted and classified into isotope-labeled and unlabeled residues (Fig. 4A). More than 95% of arginines and lysines were identified as isotope-labeled in the human protein-matched PSMs from all the database searches. In the PSMs of the HBDB search, 70.6% of arginines, 21.3% of lysines and 4.3% of prolines in the bovine protein-matched PSMs were identified as isotope-labeled, which resulted in identification of 20 bovine proteins with all isotope-labeled peptides (Fig. 4B and S4 Table). Since only newly synthesized proteins are labeled in SILAC medium, this phenomenon that FBS proteins were identified as labeled is very unlikely and suggests that improperly large databases generate increased false-positives as evidenced in Fig. 2B. All three kinds of residues were identified as unlabeled in the bovine protein-matched PSMs of the HFDB search (Fig. 4A), and all proteins identified as human contained at least one labeled peptide (Fig. 4B). Unlike the HFDB search result, the HuDB search identified an unlabeled human protein. The results suggest that our HFDB discriminates true human proteins from bovine proteins very effectively. SILAC cannot be routinely performed due to complicated time-consuming procedures and high costs. The greatest advantage of our approach, in which cell secretome is analyzed with the composite HFDB database, is that it does not require SILAC experiment.

A few attempts have been made so far to clarify the ambiguity of orthologous peptides between different species and to increase the confidence of identification. Bunkenborg et al. alluded that distinguishing bovine and human peptides in cell secretome is difficult due to a substantial number of orthologous peptides [4]. However, they did not suggest methods to maximize true-positive identifications. Other informatics-based approaches have also been attempted to increase true-positive identifications. Jagtop et al. [15] provided a two-step database search method, in which they ran the same MS data through a smaller database containing only the proteins inferred from PSMs after a primary search. Although shrinking the database increased the number of high confidence PSMs, the method did not increase the number of proteins. However, our strategy of using a composite database is highly efficient for analyzing cell secretomes without the need for multistep searches.

## Conclusion

In this study, we assessed the amount of residual FBS proteins remaining in cell culture media or on the surface of culture plates. The result of the FBS profiling was a list of about 200 proteins which was used to construct a composite database for secretome analysis. To increase true-positive identification, our raw MS data were compared against the composite database consisting of all human sequences combined with the sequences obtained from FBS profiling. This search returned more reliable results than other conventional databases with respect to error probability distribution. It is clear that searching against a proper database leads to more reliable identification. Furthermore, improved results validated our strategy without the need for multifaceted experiments like SILAC. Our strategy, using a composite database, provides a straightforward process for more easily and effectively sorting contaminants. The process is also faster and more economical without requiring complex experiments. We expect our method to improve the accuracy of human secreted proteins identification and to also add value for general use.

## Supporting Information

S1 Fig Human proteins identified from HuDB and HFDB searches of Calu-1 secretome. (PPTX)

S2 Fig MS spectra matched to different sequences between HuDB and HFDB searches of Calu-1 secretome. (PPTX)

S1 Table The list of proteins identified from FBS analysis. (XLSX)

S2 Table Peptide-spectrum matches of Calu-1 secretome according to search databases. (XLSX)

S3 Table Peptide-spectrum matches of the fractionated Calu-1 secretome sample according to search databases. (XLSX)

S4 Table Bovine proteins identified from SILAC-labeled HCT-116 secretome in HBDB versus HFDB searches. (XLSX)
