# Supplementary material for: Use of Composite Protein Database including Search Result Sequences for Mass Spectrometric Analysis of Cell Secretome
Source: PLoS One. 2015 Mar 30;10(3):e0121692. doi: 10.1371/journal.pone.0121692 (PMC4378925; doi:10.1371/journal.pone.0121692)
Supplement: S1 Fig — (PPTX) [file pone.0121692.s001.pptx]

## Slide 1
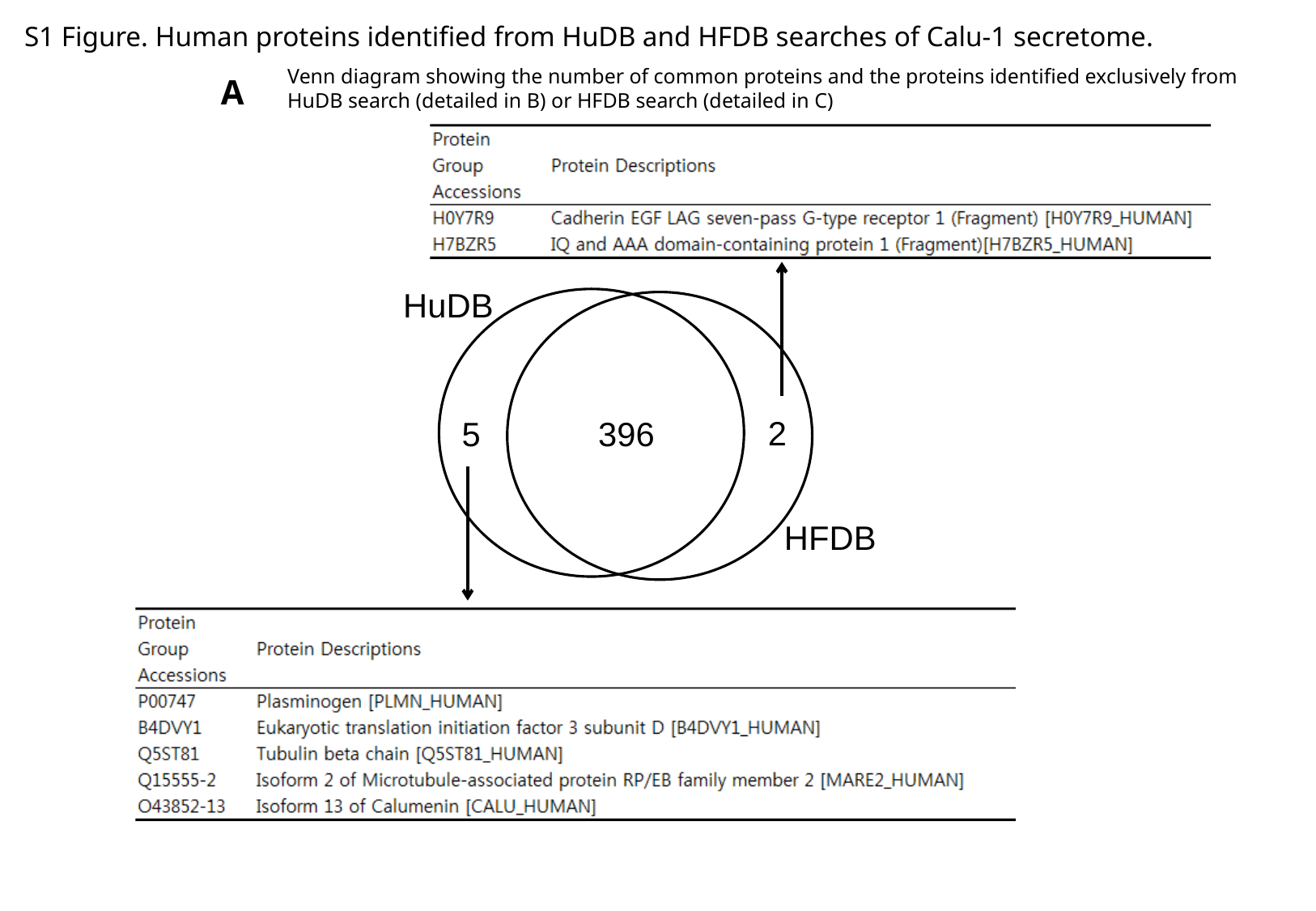

S1 Figure. Human proteins identified from HuDB and HFDB searches of Calu-1 secretome.
Venn diagram showing the number of common proteins and the proteins identified exclusively from HuDB search (detailed in B) or HFDB search (detailed in C)
A
HuDB
2
5
396
HFDB

## Slide 2
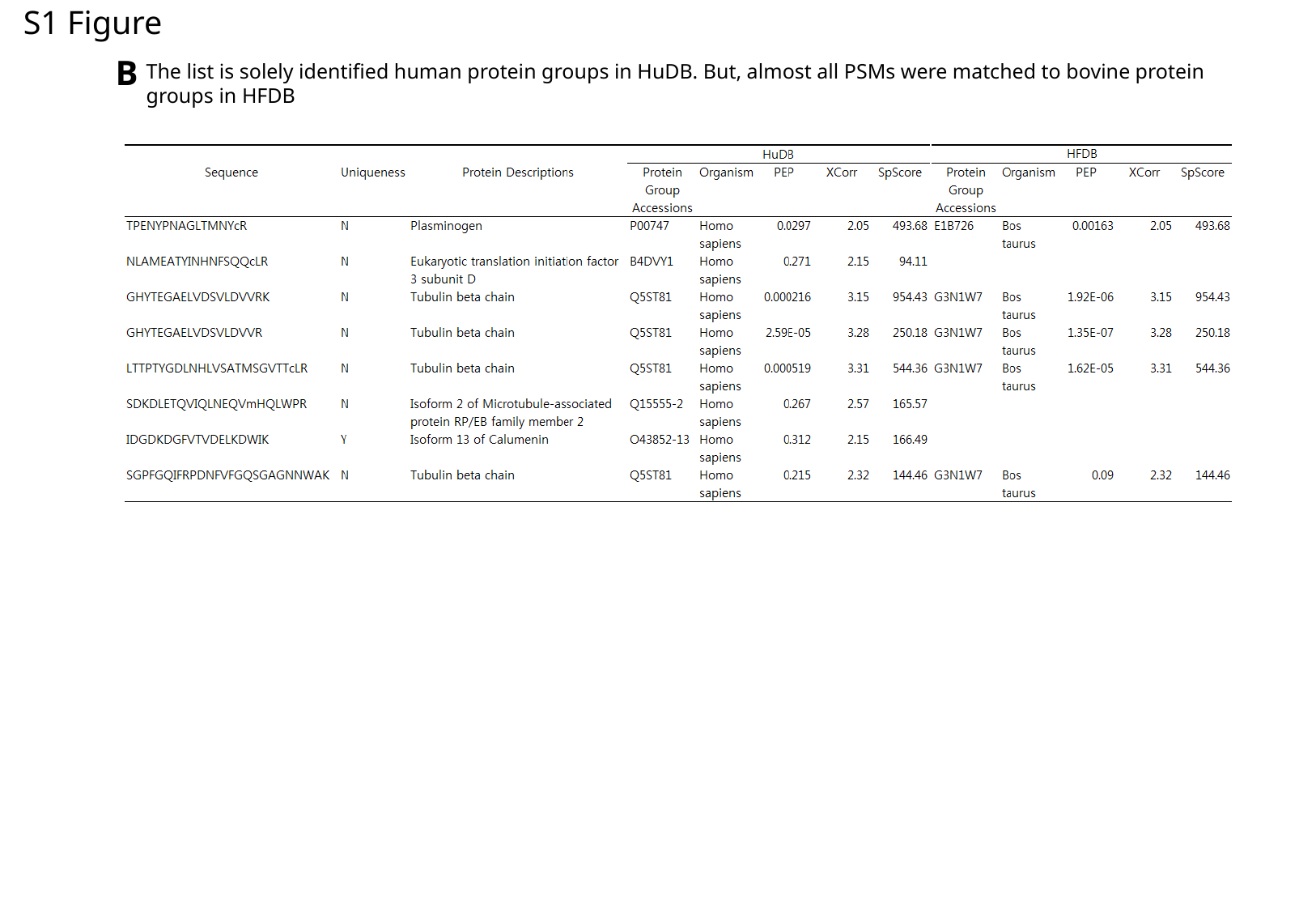

S1 Figure
B
The list is solely identified human protein groups in HuDB. But, almost all PSMs were matched to bovine protein groups in HFDB

## Slide 3
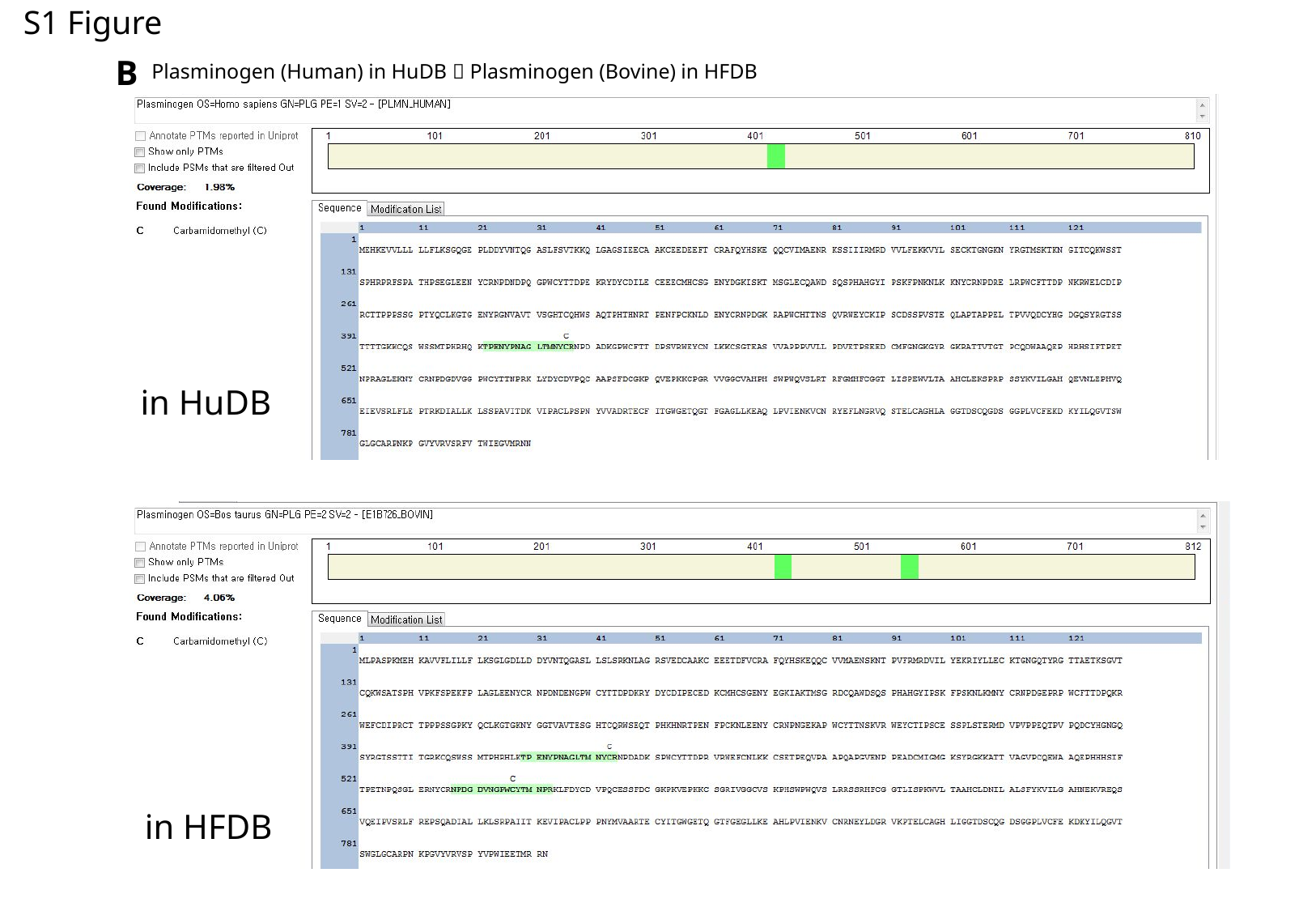

S1 Figure
B
 Plasminogen (Human) in HuDB  Plasminogen (Bovine) in HFDB
in HuDB
in HFDB

## Slide 4
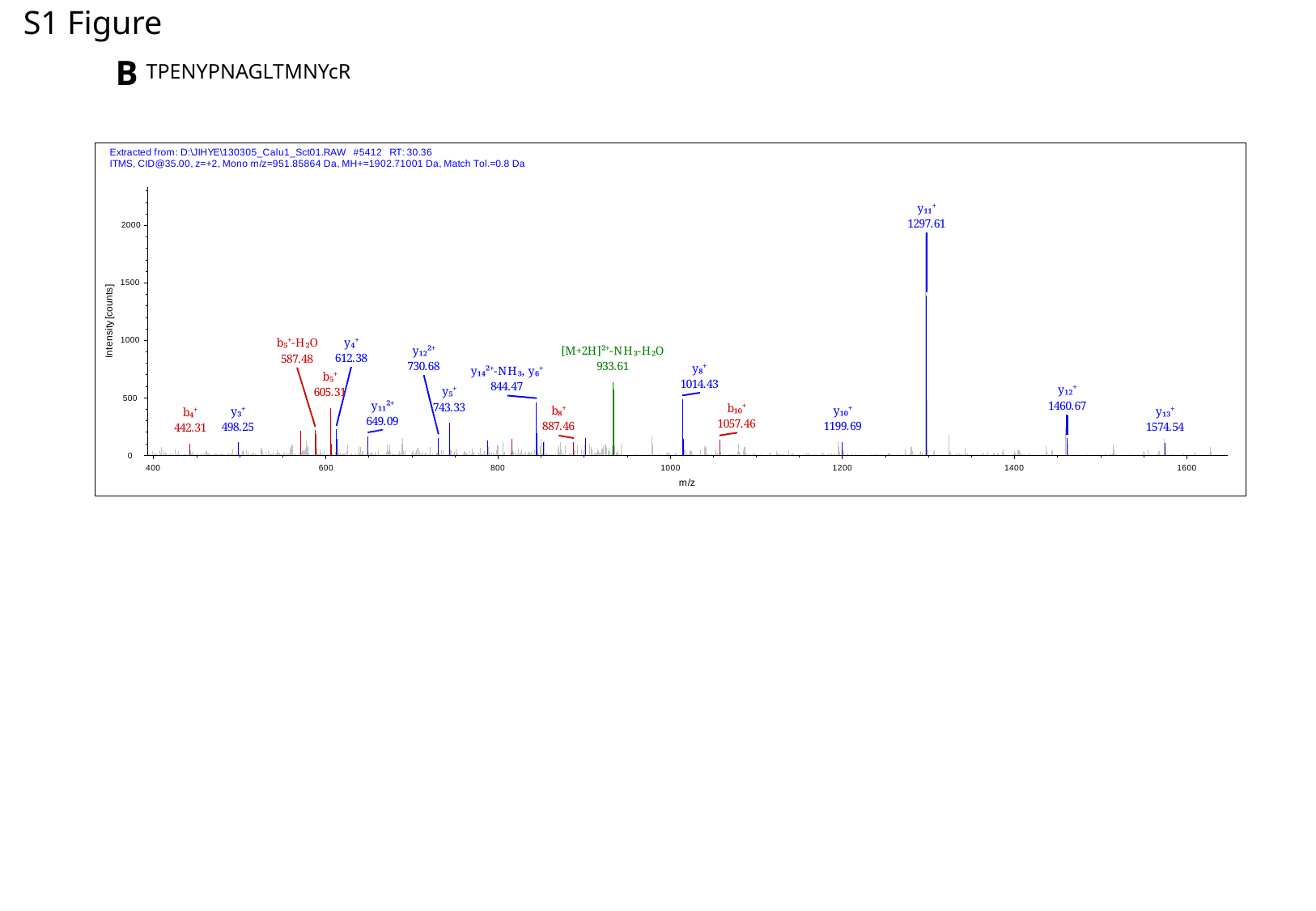

S1 Figure
B
TPENYPNAGLTMNYcR

## Slide 5
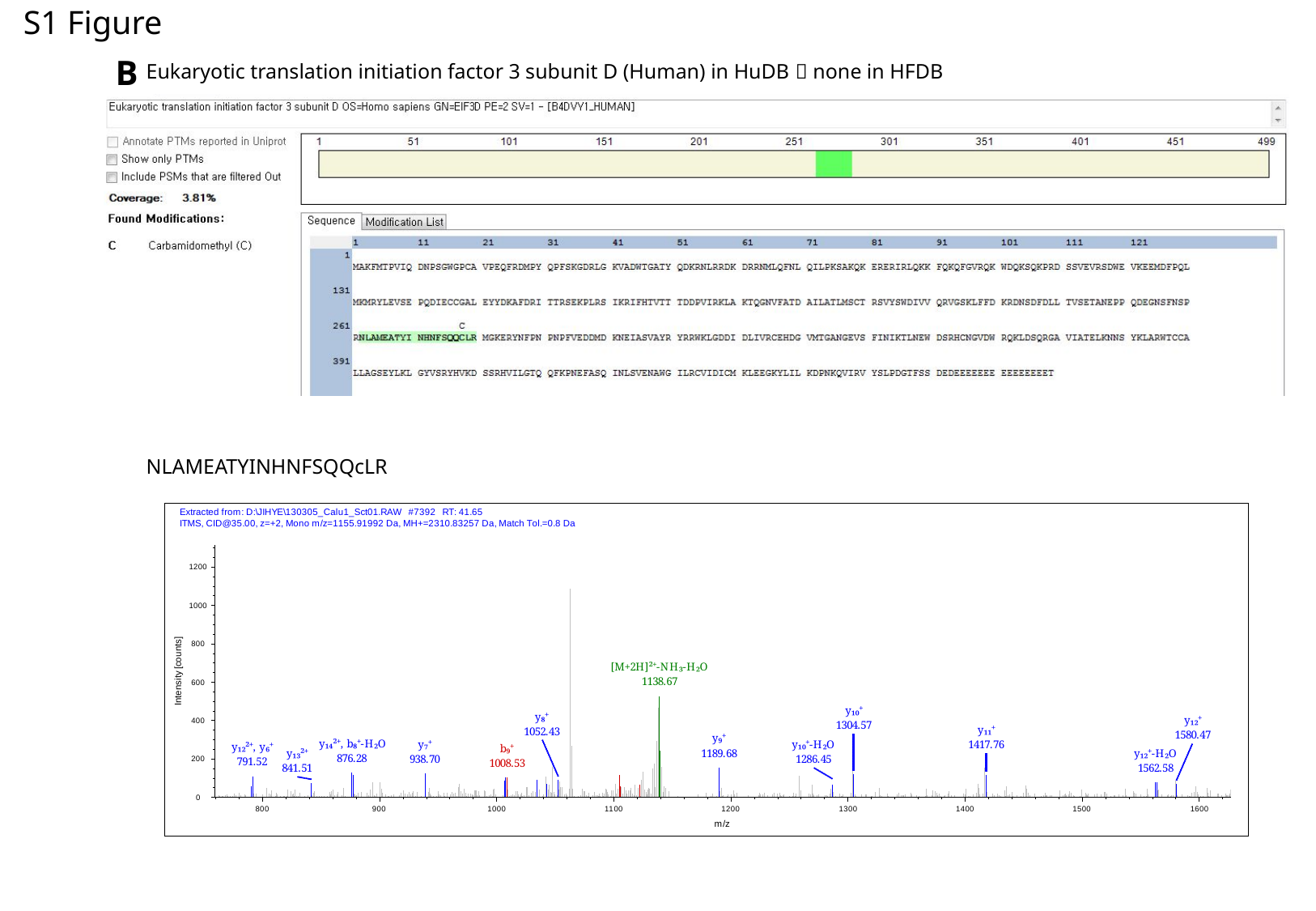

S1 Figure
B
Eukaryotic translation initiation factor 3 subunit D (Human) in HuDB  none in HFDB
NLAMEATYINHNFSQQcLR

## Slide 6
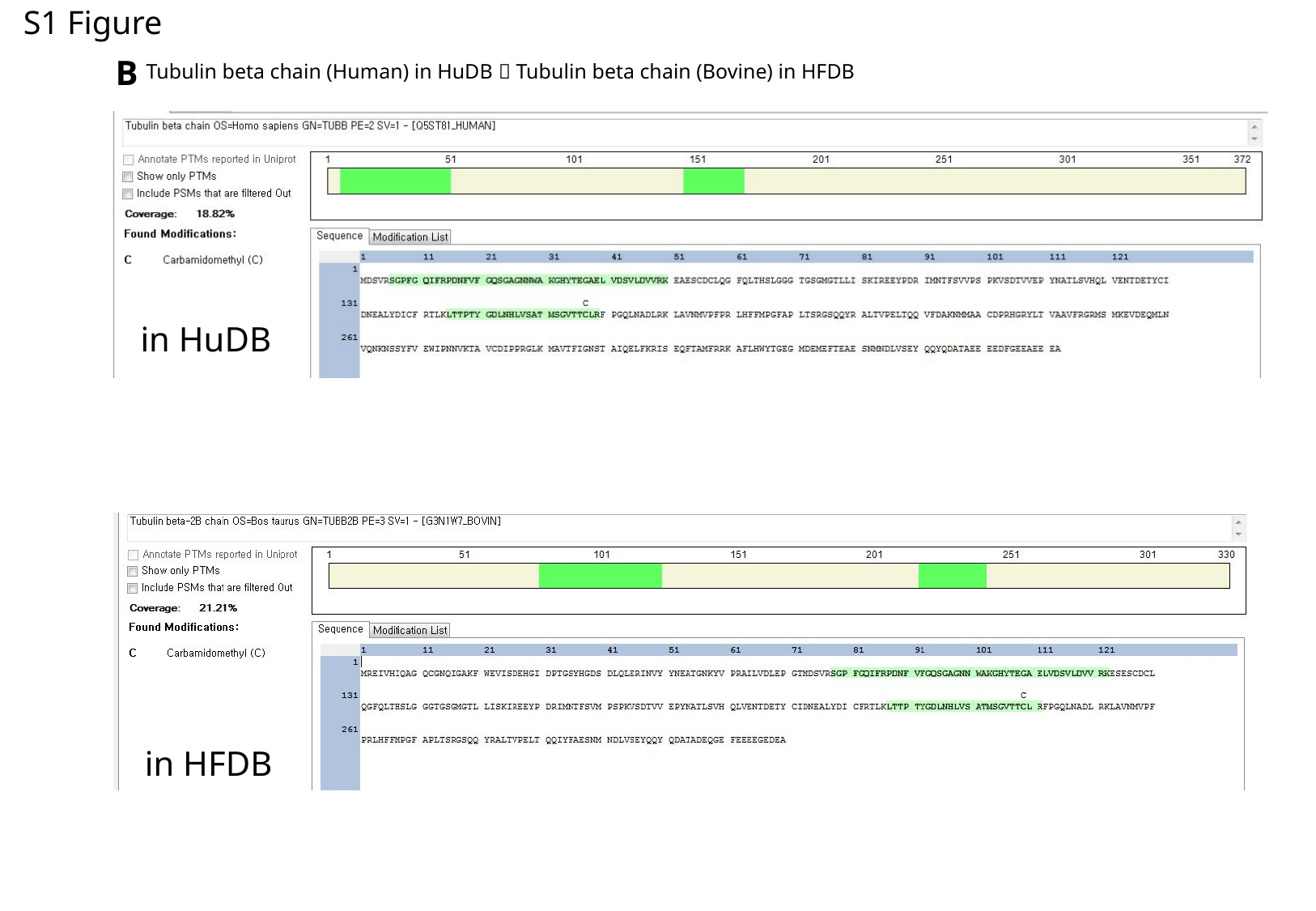

S1 Figure
B
Tubulin beta chain (Human) in HuDB  Tubulin beta chain (Bovine) in HFDB
in HuDB
in HFDB

## Slide 7
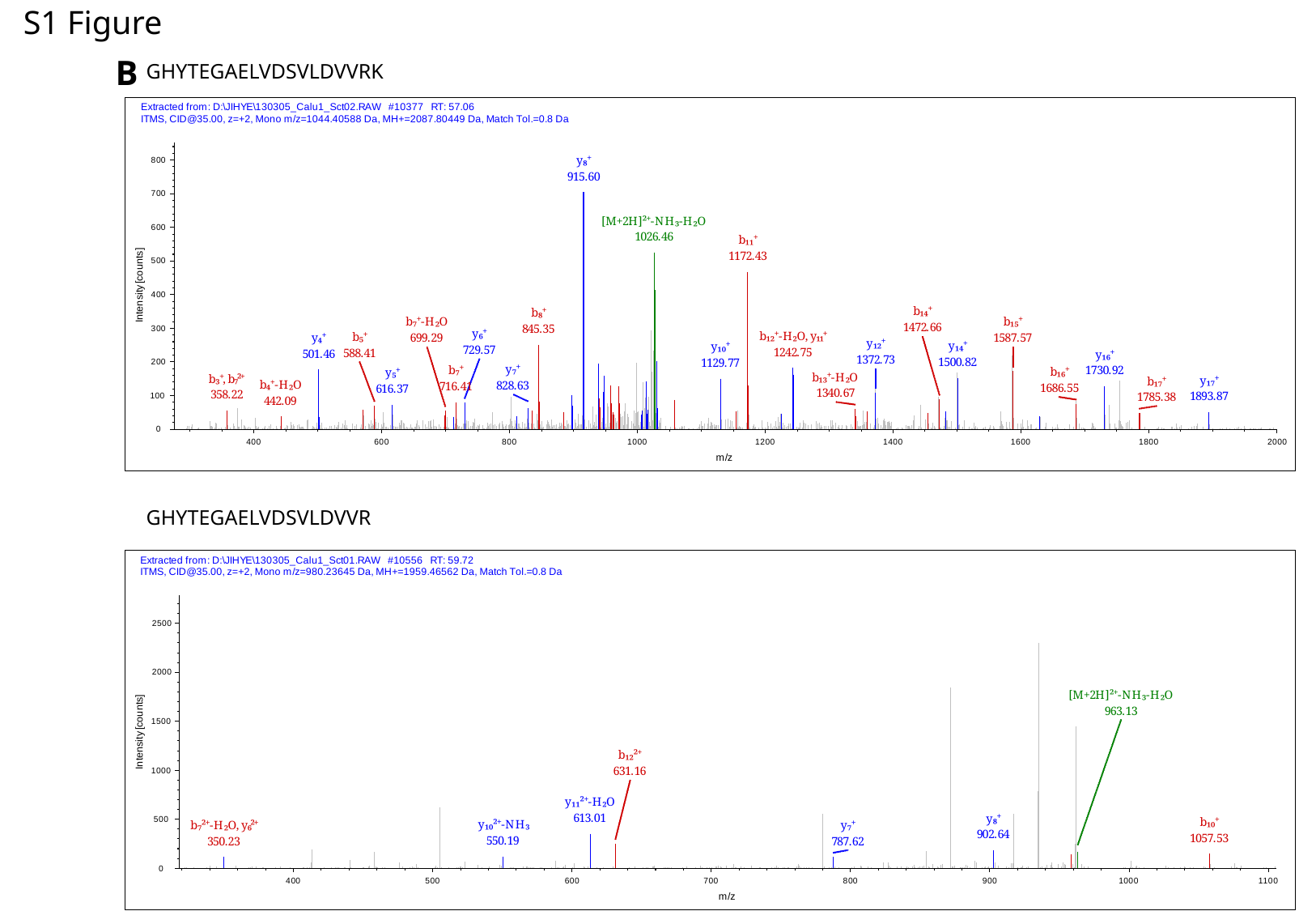

S1 Figure
B
GHYTEGAELVDSVLDVVRK
GHYTEGAELVDSVLDVVR

## Slide 8
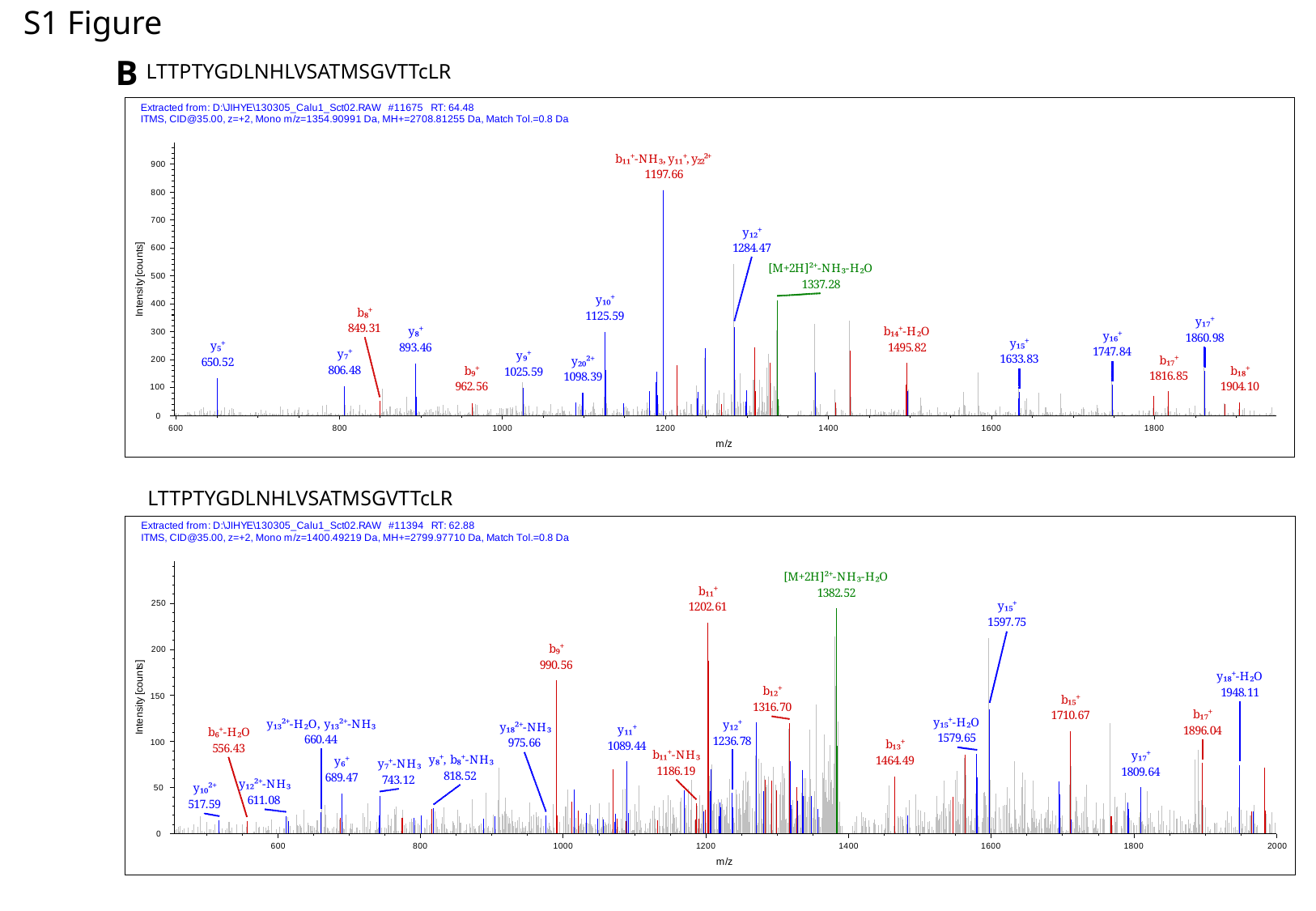

S1 Figure
B
LTTPTYGDLNHLVSATMSGVTTcLR
LTTPTYGDLNHLVSATMSGVTTcLR

## Slide 9
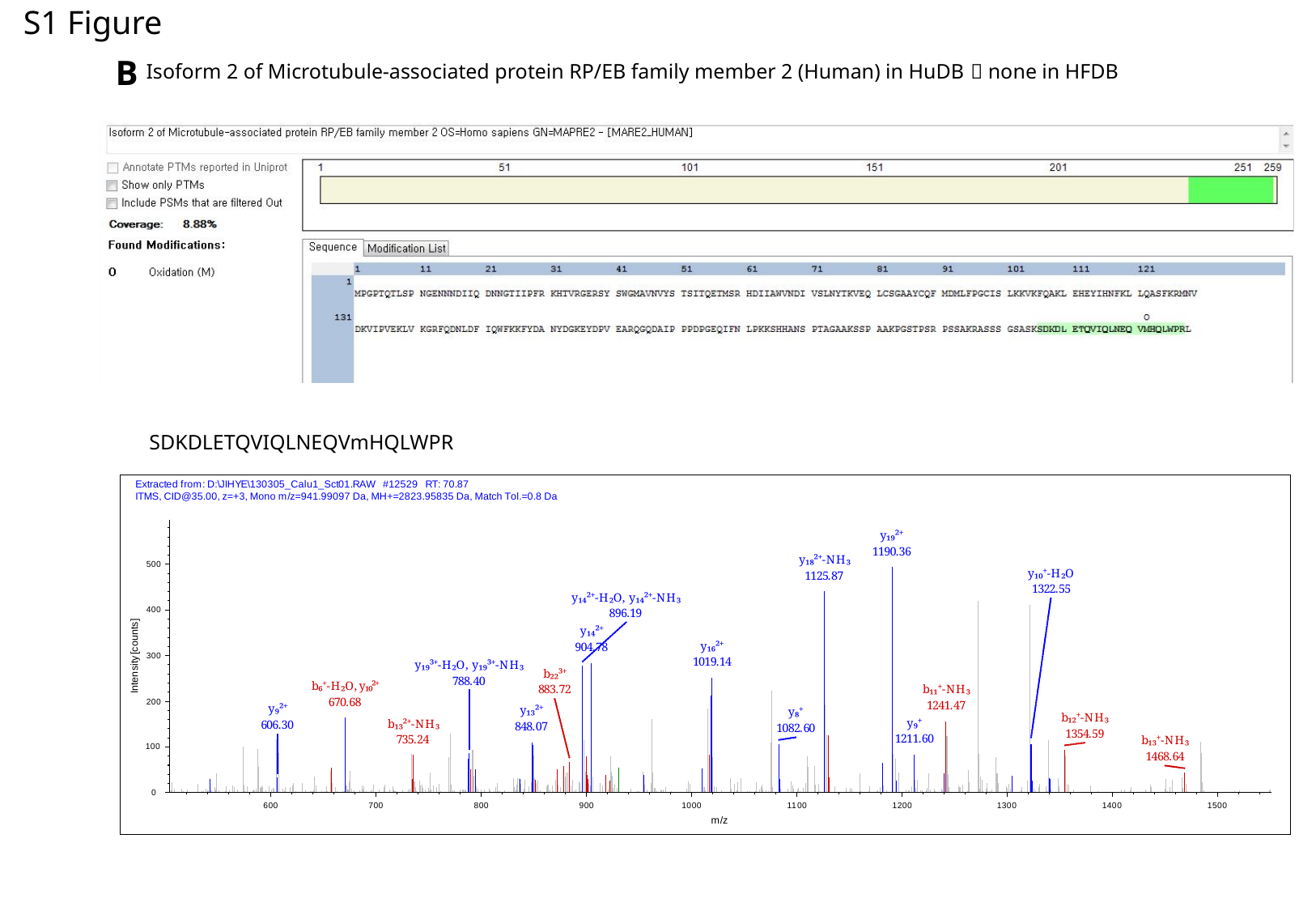

S1 Figure
B
Isoform 2 of Microtubule-associated protein RP/EB family member 2 (Human) in HuDB  none in HFDB
SDKDLETQVIQLNEQVmHQLWPR

## Slide 10
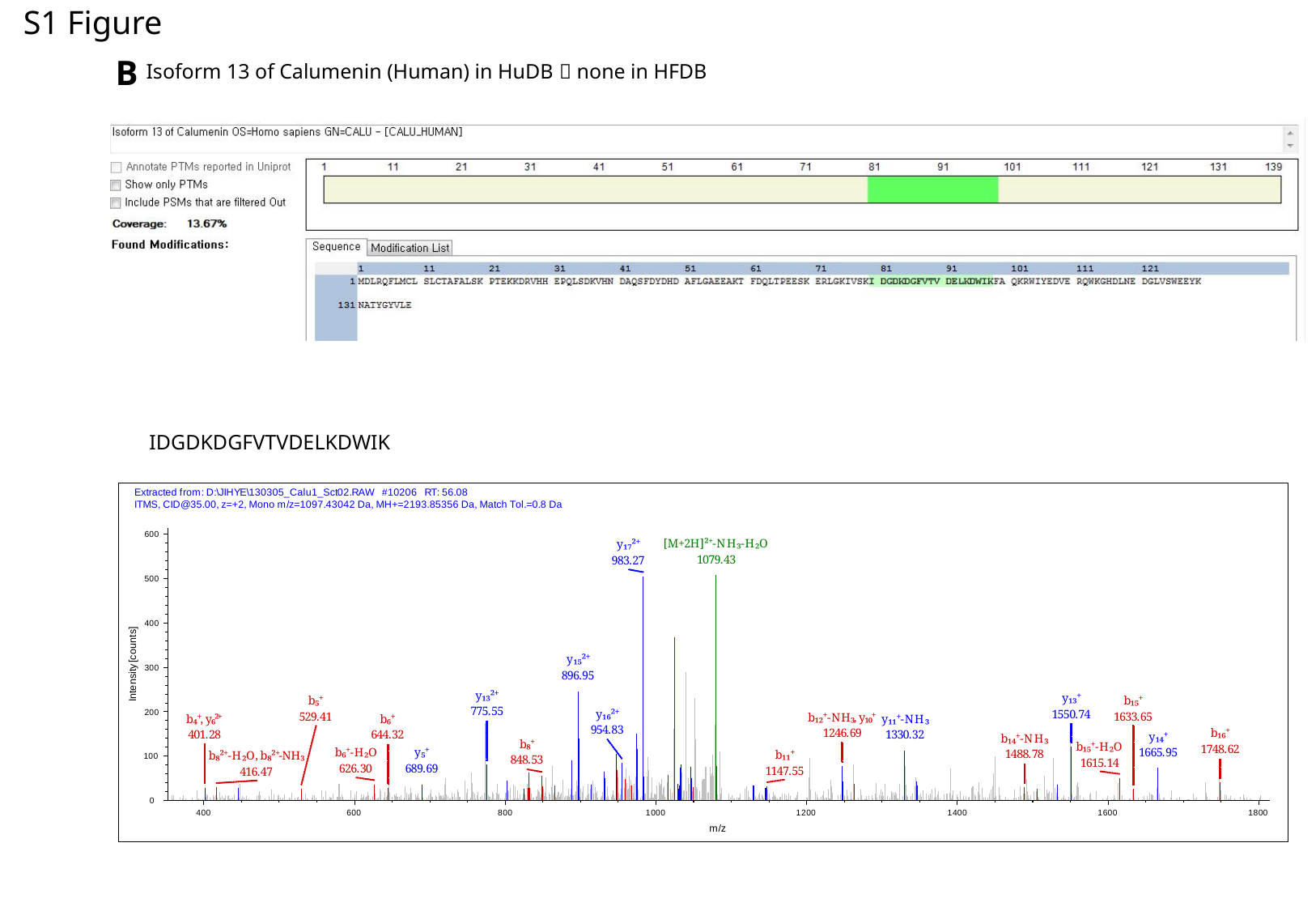

S1 Figure
B
Isoform 13 of Calumenin (Human) in HuDB  none in HFDB
IDGDKDGFVTVDELKDWIK

## Slide 11
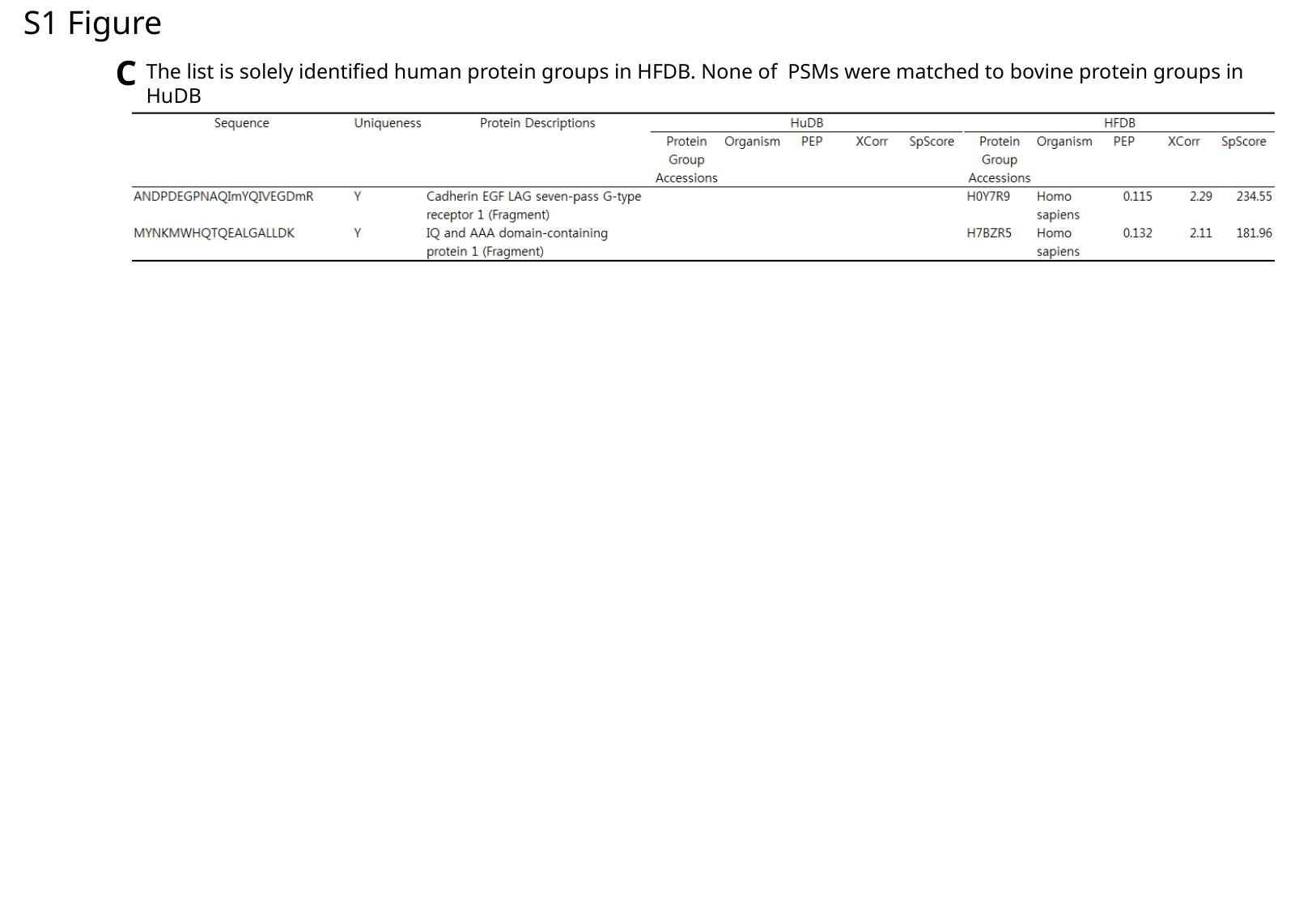

S1 Figure
C
The list is solely identified human protein groups in HFDB. None of PSMs were matched to bovine protein groups in HuDB

## Slide 12
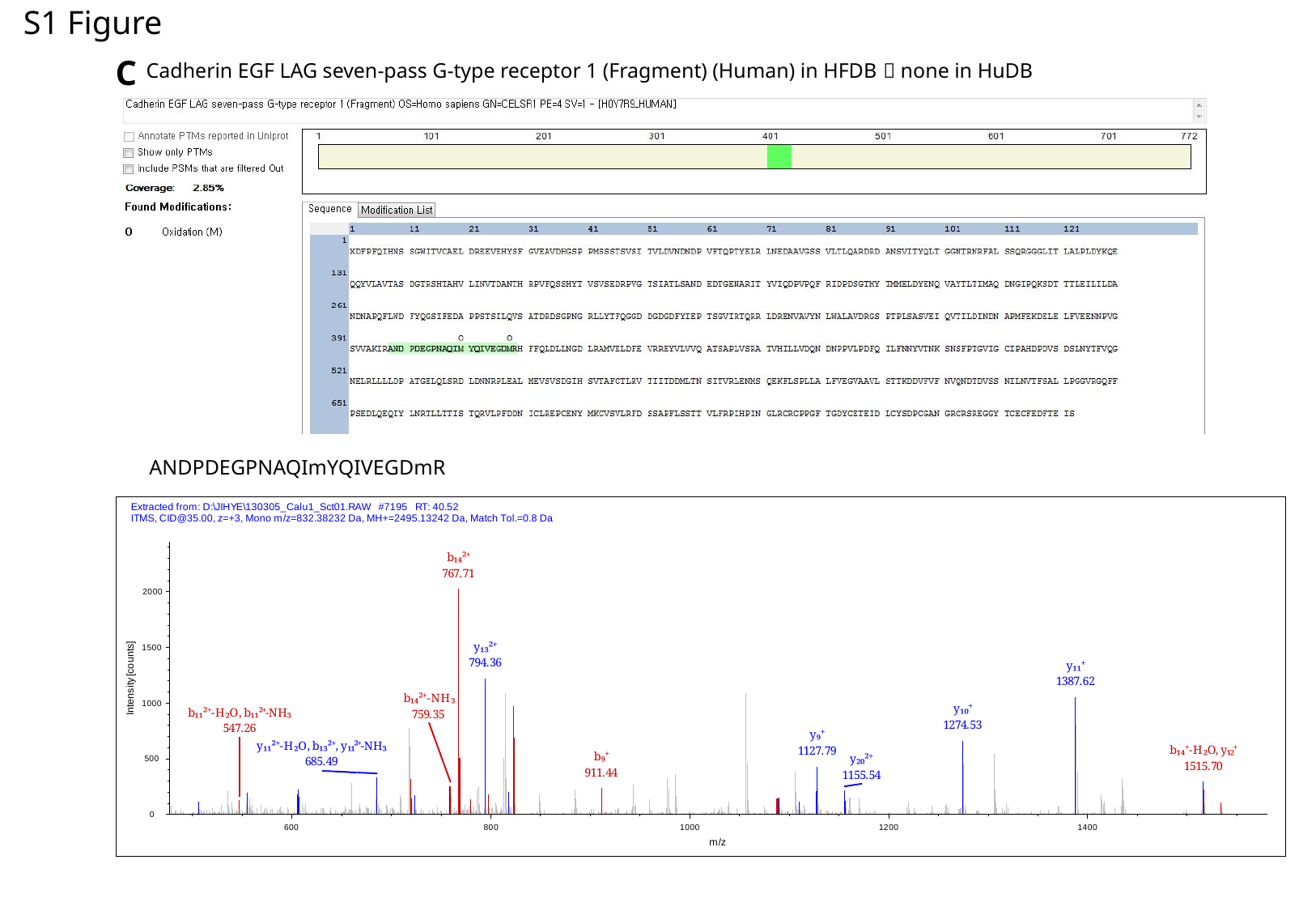

S1 Figure
C
Cadherin EGF LAG seven-pass G-type receptor 1 (Fragment) (Human) in HFDB  none in HuDB
ANDPDEGPNAQImYQIVEGDmR

## Slide 13
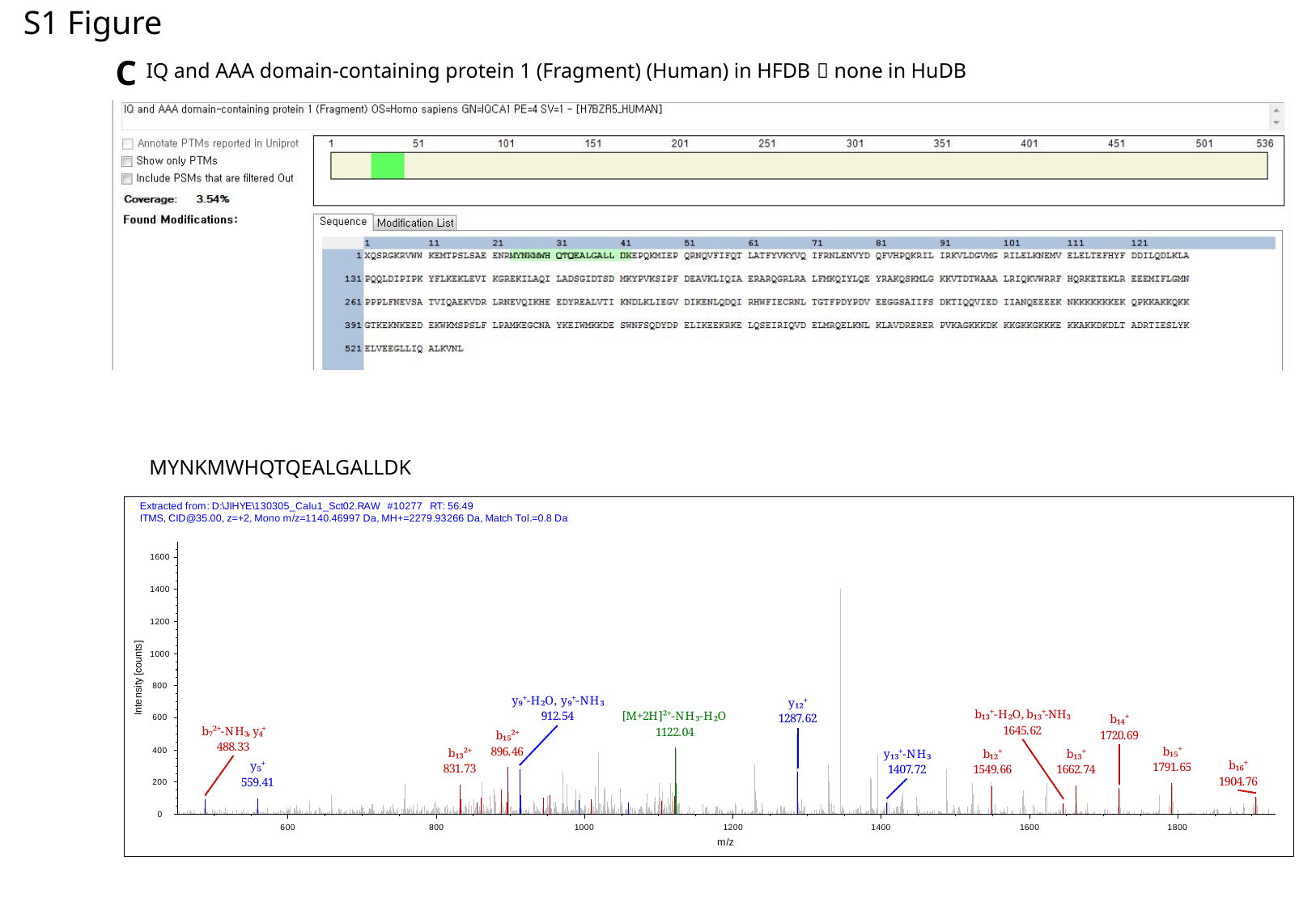

S1 Figure
C
IQ and AAA domain-containing protein 1 (Fragment) (Human) in HFDB  none in HuDB
MYNKMWHQTQEALGALLDK
