# Supplementary material for: Use of Composite Protein Database including Search Result Sequences for Mass Spectrometric Analysis of Cell Secretome
Source: PLoS One. 2015 Mar 30;10(3):e0121692. doi: 10.1371/journal.pone.0121692 (PMC4378925; doi:10.1371/journal.pone.0121692)
Supplement: S2 Fig — (PPTX) [file pone.0121692.s002.pptx]

## Slide 1
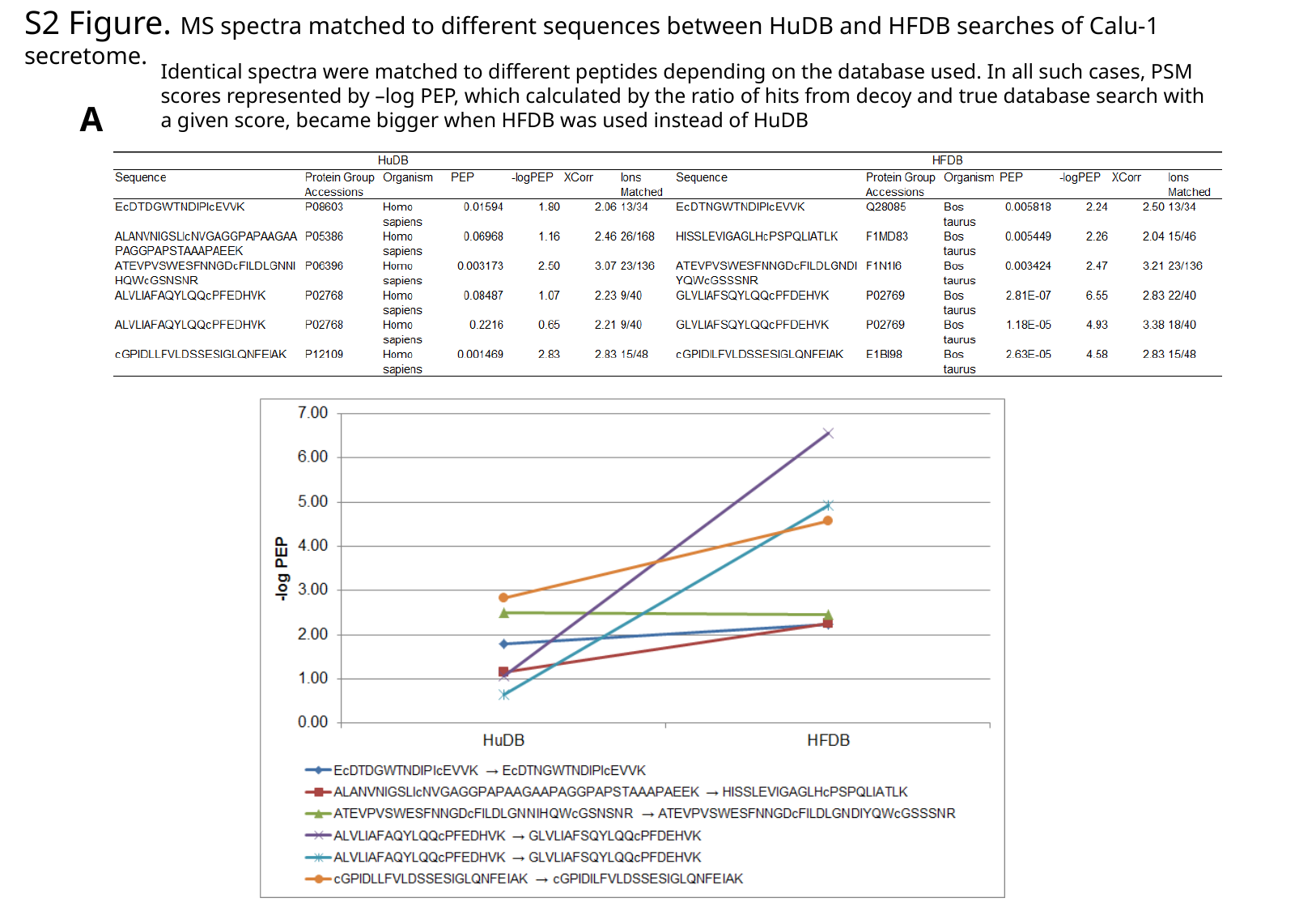

S2 Figure. MS spectra matched to different sequences between HuDB and HFDB searches of Calu-1 secretome.
Identical spectra were matched to different peptides depending on the database used. In all such cases, PSM scores represented by –log PEP, which calculated by the ratio of hits from decoy and true database search with a given score, became bigger when HFDB was used instead of HuDB
A

## Slide 2
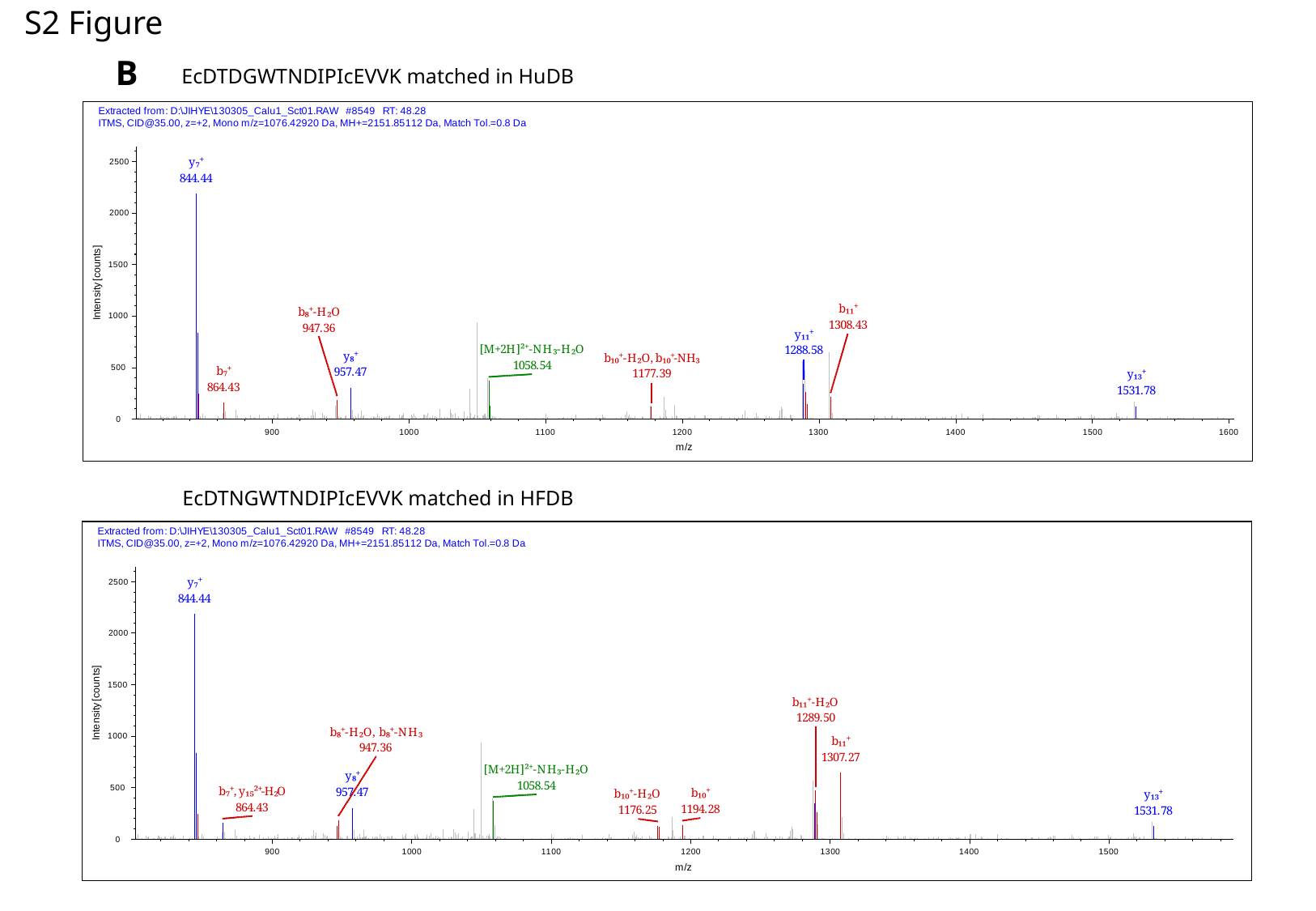

S2 Figure
B
EcDTDGWTNDIPIcEVVK matched in HuDB
EcDTNGWTNDIPIcEVVK matched in HFDB

## Slide 3
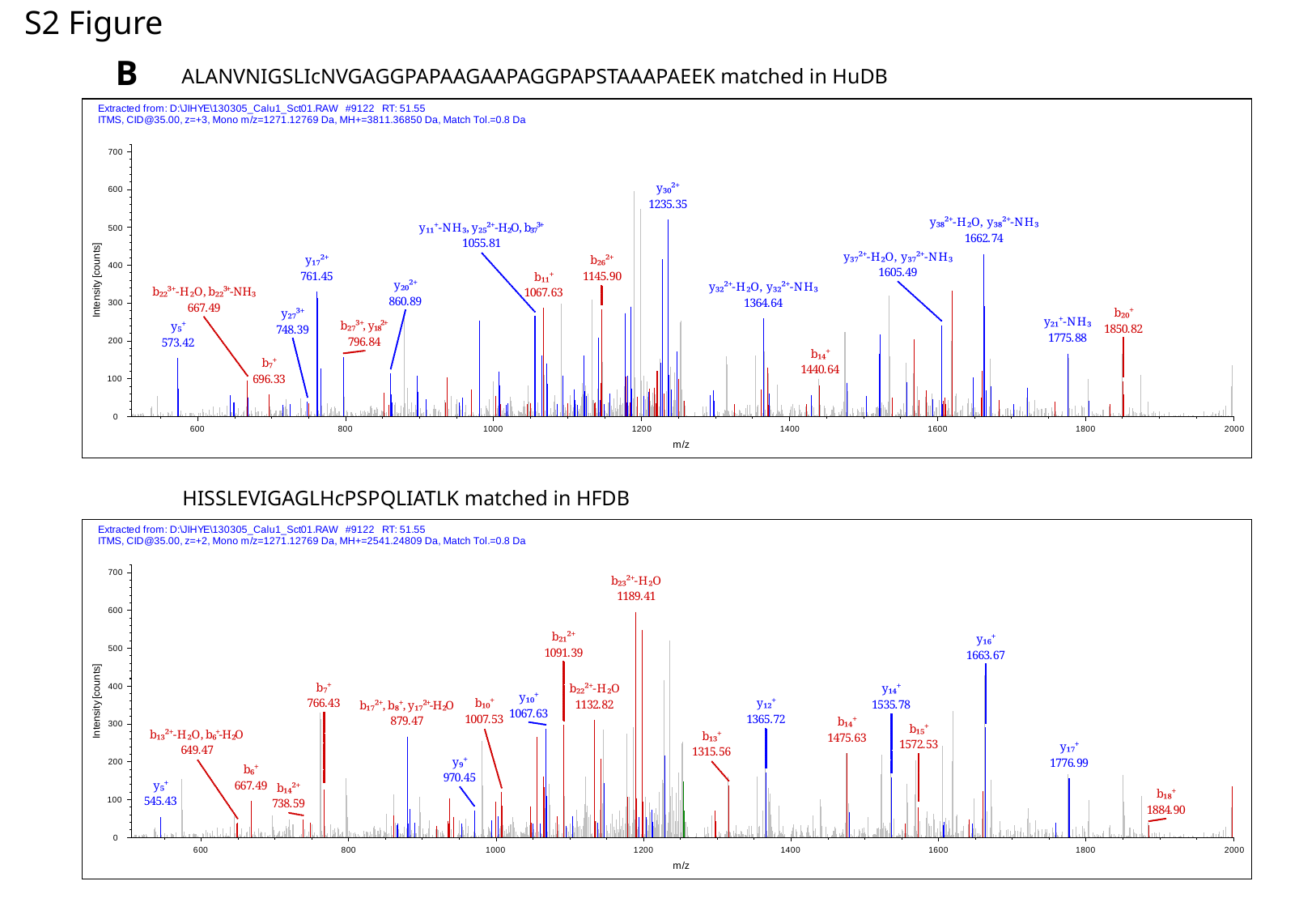

S2 Figure
B
ALANVNIGSLIcNVGAGGPAPAAGAAPAGGPAPSTAAAPAEEK matched in HuDB
HISSLEVIGAGLHcPSPQLIATLK matched in HFDB

## Slide 4
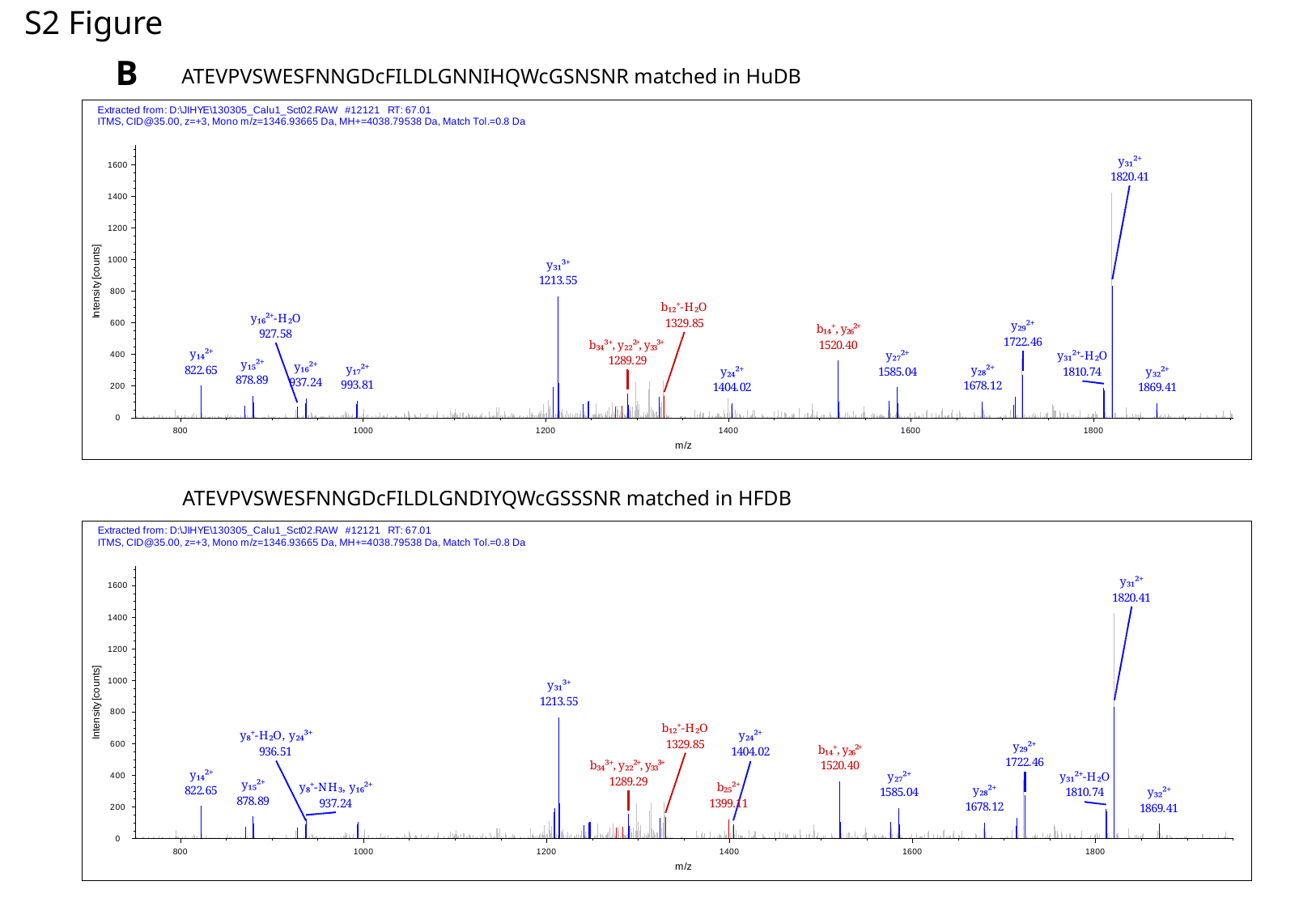

S2 Figure
B
ATEVPVSWESFNNGDcFILDLGNNIHQWcGSNSNR matched in HuDB
ATEVPVSWESFNNGDcFILDLGNDIYQWcGSSSNR matched in HFDB

## Slide 5
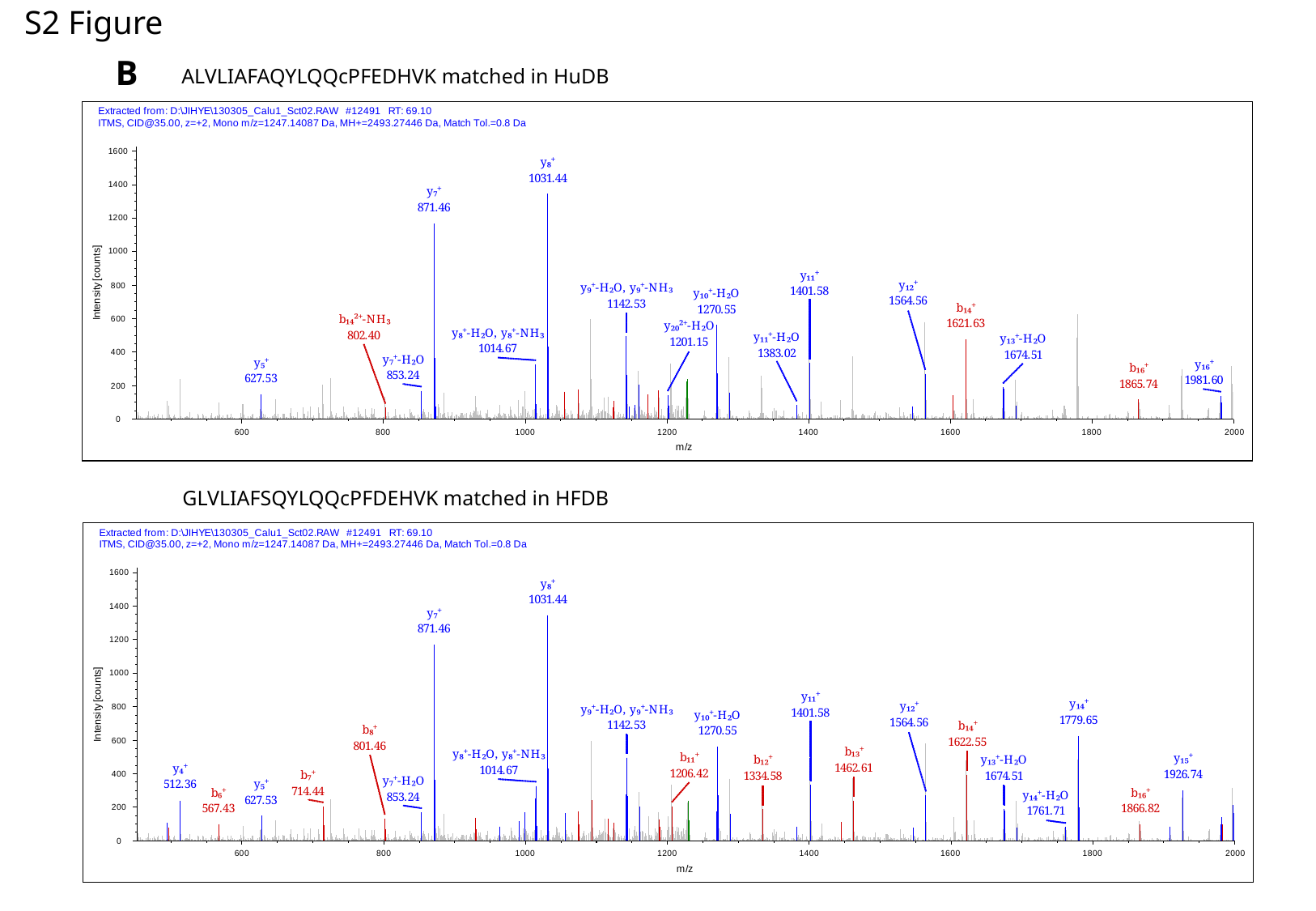

S2 Figure
B
ALVLIAFAQYLQQcPFEDHVK matched in HuDB
GLVLIAFSQYLQQcPFDEHVK matched in HFDB

## Slide 6
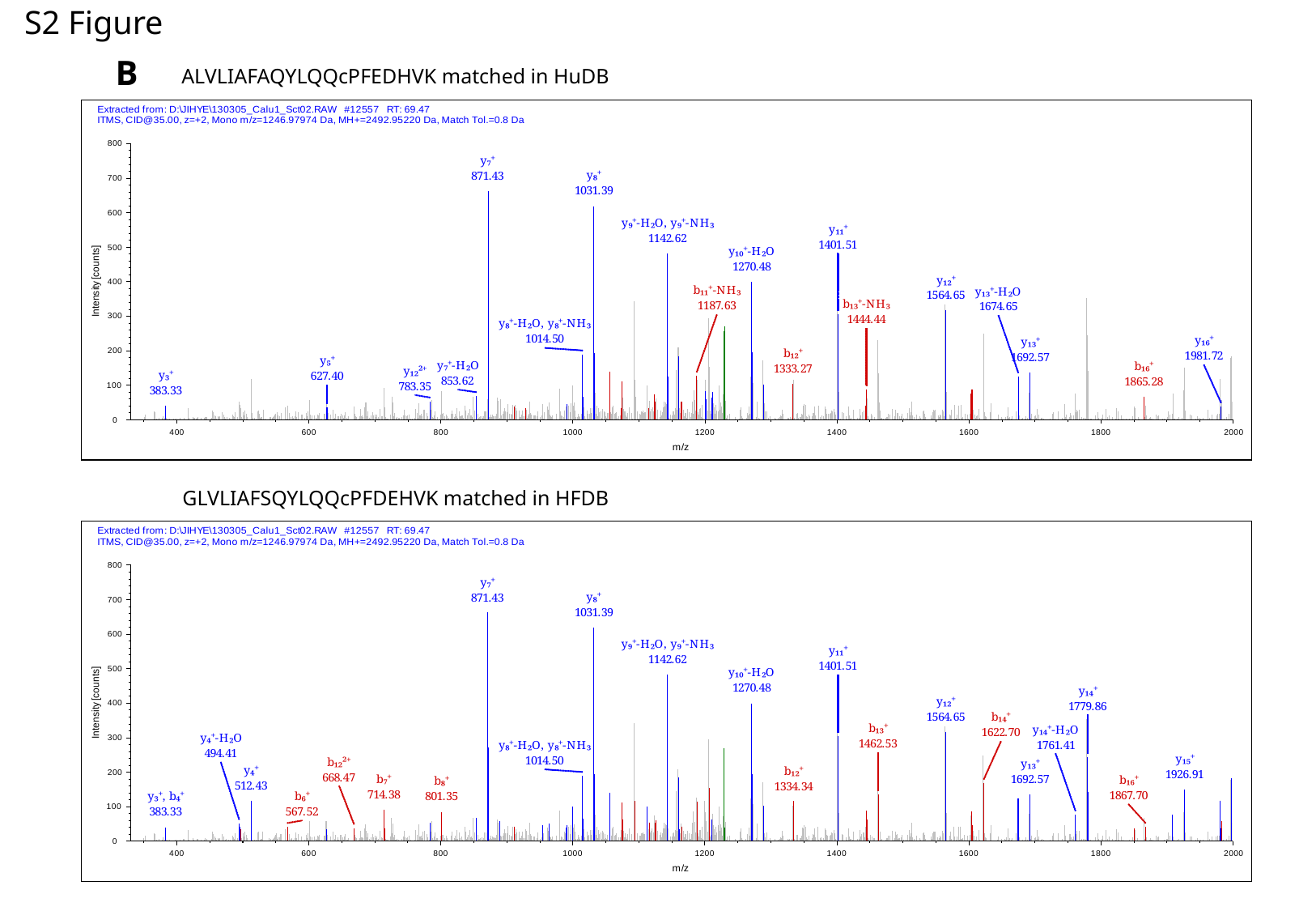

S2 Figure
B
ALVLIAFAQYLQQcPFEDHVK matched in HuDB
GLVLIAFSQYLQQcPFDEHVK matched in HFDB

## Slide 7
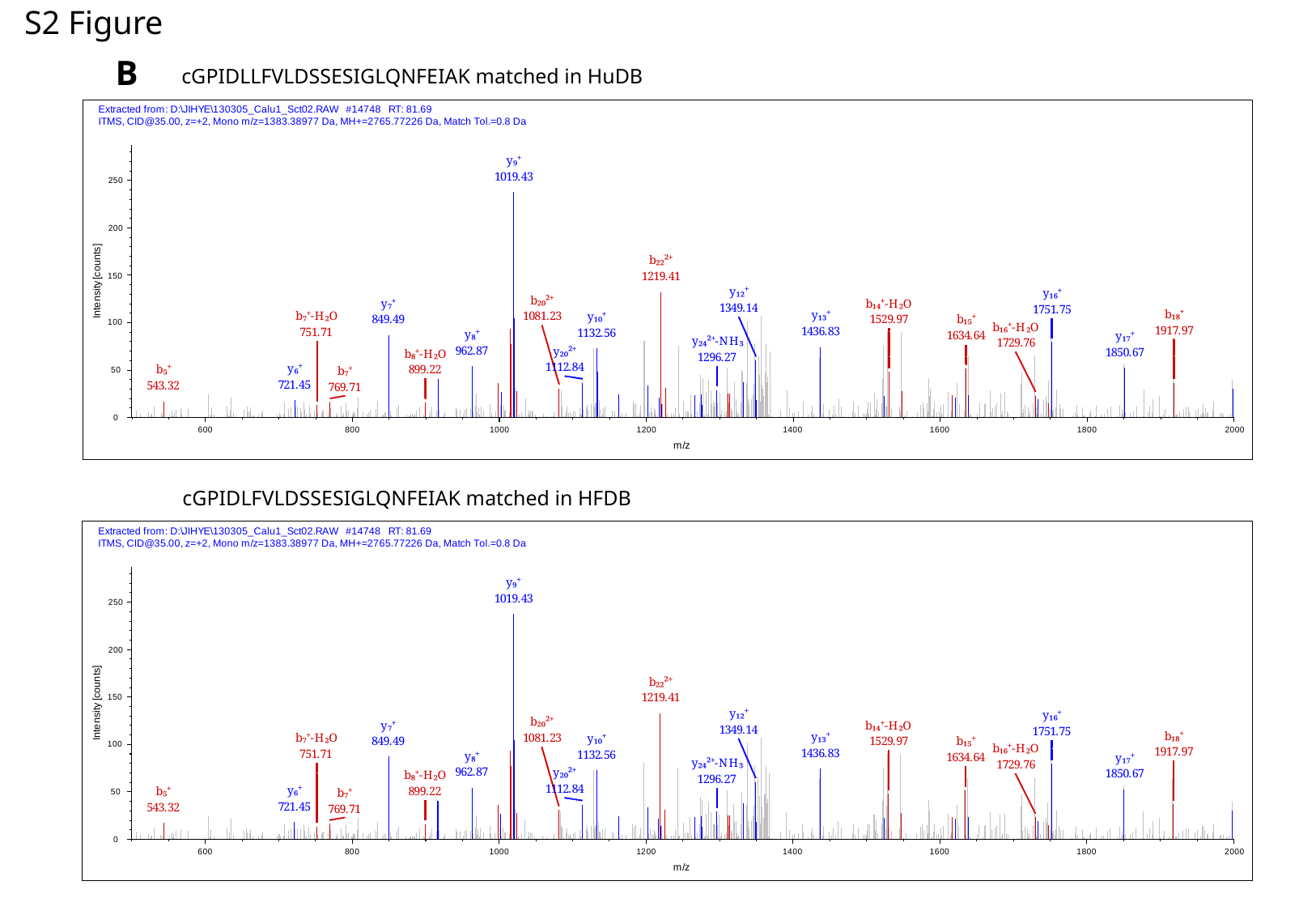

S2 Figure
B
cGPIDLLFVLDSSESIGLQNFEIAK matched in HuDB
cGPIDLFVLDSSESIGLQNFEIAK matched in HFDB
